# CystAnalyser: A new software tool for the automatic detection and quantification of cysts in Polycystic Kidney and Liver Disease, and other cystic disorders

**DOI:** 10.1371/journal.pcbi.1008337

**Published:** 2020-10-22

**Authors:** Adrián Cordido, Eva Cernadas, Manuel Fernández-Delgado, Miguel A. García-González

**Affiliations:** 1 Grupo de Xenética e Bioloxía do Desenvolvemento das Enfermidades Renais, Laboratorio de Nefroloxía (No. 11), Instituto de Investigación Sanitaria de Santiago (IDIS), Complexo Hospitalario de Santiago de Compostela (CHUS), Santiago de Compostela, Spain; 2 Grupo de Medicina Xenómica, Complexo Hospitalario de Santiago de Compostela (CHUS), Santiago de Compostela, Spain; 3 RedInRen RETIC, ISCIII, Spain; 4 Centro Singular de Investigación en Tecnoloxías Intelixentes da USC (CiTIUS) Universidade de Santiago de Compostela, Rúa Xenaro de la Fuente Domínguez, Santiago de Compostela, Spain; 5 Fundación Pública Galega de Medicina Xenómica-SERGAS, Complexo Hospitalario de Santiago de Compostela (CHUS), Santiago de Compostela, Spain; Johns Hopkins University, UNITED STATES

## Abstract

The Polycystic Kidney Disease (PKD) is characterized by progressive renal cyst development and other extrarenal manifestation including Polycystic Liver Disease (PLD). Phenotypical characterization of animal models mimicking human diseases are commonly used, in order to, study new molecular mechanisms and identify new therapeutic approaches. The main biomarker of disease progression is total volume of kidney and liver in both human and mouse, which correlates with organ function. For this reason, the estimation of the number and area of the tissue occupied by cysts, is critical for the understanding of physiological mechanisms underlying the disease. In this regard, cystic index is a robust parameter commonly used to quantify the severity of the disease. To date, the vast majority of biomedical researchers use ImageJ as a software tool to estimate the cystic index by quantifying the cystic areas of histological images after thresholding. This tool has imitations of being inaccurate, largely due to incorrectly identifying non-cystic regions. We have developed a new software, named CystAnalyser (register by Universidade de Santiago de Compostela–USC, and Fundación Investigación Sanitaria de Santiago—FIDIS), that combines automatic image processing with a graphical user friendly interface that allows investigators to oversee and easily correct the image processing before quantification. CystAnalyser was able to generate a cystic profile including cystic index, number of cysts and cyst size. In order to test the CystAnalyser software, 795 cystic kidney, and liver histological images were analyzed. Using CystAnalyser there were no differences calculating cystic index automatically versus user input, except in specific circumstances where it was necessary for the user to distinguish between mildly cystic from non-cystic regions. The sensitivity and specificity of the number of cysts detected by the automatic quantification depends on the type of organ and cystic severity, with values 76.84–78.59% and 76.96–89.66% for the kidney and 87.29–93.80% and 63.42–86.07% for the liver. CystAnalyser, in addition, provides a new tool for estimating the number of cysts and a more specific measure of the cystic index than ImageJ. This study proposes CystAnalyser is a new robust and freely downloadable software tool for analyzing the severity of disease by quantifying histological images of cystic organs for routine biomedical research. CystAnalyser can be downloaded from https://citius.usc.es/transferencia/software/cystanalyser (for Windows and Linux) for research purposes.

## Author summary

This work suggests CystAnalyser is the most reliable software tool currently available for the assessment of cystic pathologies including Polycystic Kidney Disease (PKD) and Polycystic Liver Disease (PLD). CystAnalyser combines automatic cyst recognition with a friendly graphical user interface, allowing user input prior to histological image quantification. CystAnalyser responds to the need to obtain reliable measurements of the universal biomarker for PKD and PLD disease progression, the Cystic index (area of cysts within the total area of tissue). This software tool is also able to calculate the number and size of cysts from the histological images. In summary, our results show that CystAnalyser overcomes the precision issues detected using the most commonly used software to date (ImageJ) for Cystic index quantification, offering users a reliable tool to easily characterize the phenotype and the pathophysiology of PKD and PLD in pre-clinical studies using animal models.

This is a *PLOS Computational Biology* Software paper.

## Introduction

Autosomal Dominant and Autosomal Recessive Polycystic Kidney Disease (ADPKD and ARPKD, respectively) are the most common forms of inherited cystic disorders. Mutations in *PKD1* and *PKD2* genes are the main cause of ADPKD, while mutations in *PKHD1* gene are responsible for ARPKD [1]. Polycystic Liver Disease (PLD) is the most frequent extra-renal manifestation of ADPKD, but it can be also found as an isolated autosomal dominant form of Polycystic Liver Disease (ADPLD) [2]. PKD and PLD are characterized by the progressive replacement of the renal and hepatic parenchyma with cysts of varying size, associated with a gradual increase in renal and liver volume [3,4]. Both diseases have a broad spectrum of severity, but in general PKD is associated with a much more severe phenotype than PLD [5,6]. Cyst initiation or cystogenesis mostly occurs at embryonic stage, with increasing number of cysts and cystic volume expansion with the age [7,8], ultimately leading to renal and/or liver transplantation [9,10]. It has been established that the principal biomarker to provide a metric progression of the disease and monitor treatment efficacy is the measurement of total kidney and liver volume by imaging technologies [11,12]. We and other researchers have developed several *Pkd1*, *Pkd2* or *Pkhd1* animal models to study the pathological mechanisms undergoing cyst formation and progression including identifying genetic interactions and testing new therapeutic approaches [13–16]. Although magnetic resonance imaging (MRI) or sonographic images have been the standard methods for measuring the progression of PKD or PLD in humans, digital and histological microscopy images have been the most commonly used in translational research. For these studies, it is well established that the analysis of the cumulative area of cysts within the total area of the kidney, liver or other organs (Cystic Index or CI) as a measurable biomarker to quantify cystogenesis, cyst expansion and to track the severity and progression of the disease.

The most commonly used tool to calculate the cystic index from histological samples is ImageJ, a multiplatform free software developed in the Java programming language [17,18,27–29,19–26]. This software develops an image thresholding which creates a binary image, where the cystic index is computed with the white regions defined as cysts and the black regions as tissue (background). However, it is known to researchers that this value is imprecise because many of the white regions in the image (lumen of arteries, veins, or non-cystic regions) are mistakenly considered to be cysts. In addition, ImageJ does not allow reviewing of the segmentation before the calculation process or the quantification of the cysts based on their profile. Therefore, a more precise tool is needed to quantify the cystic index in a reliable and accurate manner. Recently, our group has developed the software Govocitos to estimate the fecundity of fish from histological images of its gonads by combining automatic processing of the image with a friendly Graphical User Interface (GUI) to review the process before quantification [30,31]. Based on scientific needs in the PKD and PLD fields, we have generated CystAnalyser as a software tool for the quantitative analysis of histological images from cystic kidney and liver samples, in order to reliably and accurately quantify their cystic index, cyst number and cyst size. We have developed sophisticated image analysis and machine learning algorithms to automatically recognize and quantify image data, and then compared our software with ImageJ. In addition, to being easier to use by individuals new to the field of research, CystAnalyser has proven to be significantly more sensitive and reliable, and less subjective, in quantifying the cystic area, cyst number and size, than the most commonly used software in cystic disease research.

## Results

### Description and functionality of CystAnalyser

**Fig 1:**
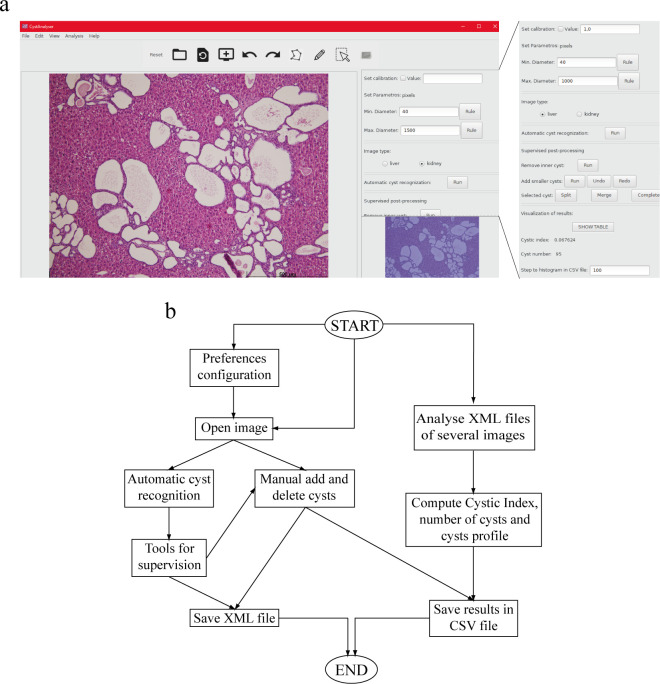
CystAnalyser overview. (a) Main window of CystAnalyser with a histological image of cystic liver (as example). On the right, we can see the full lateral panel showing the available *Automatic cyst recognition*, *Supervised post-processing* and *Visualization of Results* tools. (b) A flowchart containing the main tasks and functions of CystAnalyser. For more details and information see “Description and Functionality of CystAnalyser” sub-section (in “Results” section).

CystAnalyser is written in the C++ programming language using the GTK+ (“GIMP Tool Kit”) library (https://www.gtk.org) to develop the GUI, and the C++ interface of the OpenCV library (https://opencv.org) to process and recognize cysts in the image. Fig 1 shows the main window of CystAnalyser with the lateral panel and a flowchart describing the main functionality for the user. CystAnalyser can work with default or preset configurations, allowing to set image preferences like image/ file paths, calibration, working colors, shapes drawn on the image and the type of tissue to be analyzed (liver or kidney) and saved for future analysis. The side panel contains all these settings as well as *Automatic cyst recognition*, *Supervised post-processing* and *Visualization of Results* tools. By clicking the *Run* button at the *Automatic cyst recognition* tool, cysts are recognized and drawn on the image. The user can review or modify the automatic cyst detection by using the following setup from the *Supervised post-processing* tool: 1) *Remove inner cyst* button: click on the button *Run* after the label *Remove inner cyst* to remove the automatically detected cysts, which fall into some cysts manually drawn by the user; 2) *Add smaller cysts* button: click on the *Run* button to automatically add smaller cysts, this functionality is oriented only to use with very small cysts that are not recognized by the *Automatic cyst recognition* tool. *Undo*/*Redo* can be used to include or exclude this analysis; 3) *Selected cyst* button: several cysts incorrectly recognized by the *Automatic cyst recognition* tool as only one cyst can be split consecutively by using the *Split* button. On the contrary, when one cyst is incorrectly recognized by *Automatic cyst recognition* as several cysts, they can be fused with the *Merge* tool. The zoom tool allows users to get very precise detail by magnifying a selected part of the image and manually draw a cyst or delete a selected cyst on the image.

The *Visualization of results* tool automatically shows the cystic index and number of cysts, allowing the user to explore the size of each drawn cyst in real time or to display a table with specific information for all cysts by clicking the *SHOW TABLE* tool (the cysts can be highlighted in the image by selecting them in the table). In addition, CystAnalyser allows the user to download and upload results in XML (eXtensible Markup Language) and CSV (Comma-Separated Values) format files. The CVS file contains statistical information provided by quantitative analysis of the images: 1) spatial resolution, i.e. the relation between micrometers and pixels, at which the images were digitized, also known as calibration, which must be pre-specified by the user; 2) image size in pixels; 3) cystic index; 4) number of cysts; and 5) a profile of the cystic sizes, i.e. the number of cysts for each range of diameters or areas. A user guide has been provided for detailed description (S1 Text).

### Development of algorithms for automatic cyst recognition

The algorithms proposed to recognize the cysts on the image are a combination of known techniques in the computer vision field [32], implemented using the OpenCV modules *core* and *imgproc*. Firstly, the image has been segmented to transform it into a binary image, where white and black colors represent a cyst and the background, respectively. Secondly, this binary image was analysed in order to extract the contours of the cysts. The main algorithmic steps are: 1) extract green channel (IG) from the original RGB image; 2) compute the average grey level (μG) from IG; 3) threshold the image IG using the level μG, resulting in a binary image IB; 4) apply the kmeans clustering algorithm [33], implemented by the OpenCV *kmeans*, to IG using as initialization the image IB and two clusters, resulting in a binary image Ik, which normally has spurious noise; 5) post-process the image Ik with mathematical morphology in order to remove the smaller regions with noise resulting in another binary image Io; and 6) extract the contour of the cysts from Io using the algorithm proposed by Suzuki and Be [34], which was implemented by the OpenCV function *findContours*. To provide the minimum and maximum cystic diameters (dmin and dmax), the user must manually specify the diameter or draw a diameter line on the image using the CystAnalyser graphical user interface. Finally, a size filter was applied to remove the regions with diameters outside the interested interval [dmin, dmax]. All the algorithmic steps were equal for both kidney and liver images, except in post-processing (step 5), where the mask size of the open morphological filter was constant and equal to three pixels for all liver images so as to remove noise in the binary image, *I*_*k*_. This filter was parametrized by d_min_ for kidney images, in order to remove the objects smaller than the desired cyst.

Using a preliminary version of CystAnalyser, the automatic algorithm showed difficulties distinguishing renal tubules from very small renal cysts. We updated CystAnalyser to include a supervised classifier in order to discriminate between true cysts and false positives by activating *Add smaller cysts* setup (click on the *Run* button to automatically add smaller cysts), this functionality was oriented only to use with very small cysts initially not recognized by the *Automatic cyst recognition* tool (see lateral panel in Fig 1A). We used the Support Vector Machine (SVM) classifier [35], with radial basis function (RBF) kernel implemented by the LibSVM library [36]and accessed through its C binding. The SVM used the true data generated with the first version of the program. We used a total of 7535 small cysts (true cysts were annotated by an expert, and the false cysts were provided by CystAnalyzer during the cyst recognition process), 50% of them devoted to training, 25% to validation and 25% to test. The experimental setting used 4-fold cross validation. The selection of values for the SVM tuning hyper-parameters (the regularization parameter *C* and the inverse γ of the RBF kernel spread) used the well-known grid search method, trying values {2^*i*^} from *i* = -5 to 15, step 2 for C and {2^*i*^} from *i* = -13 to with step 2 to γ. The combination of values that achieved the highest average performance (measured by the Cohen kappa statistic) on the 4 validation sets were *C* = 2 and γ = 2^−5^. The average kappa and accuracy values over the test sets were 68.7% and 86.7%, respectively, although the performance of CystAnalyzer working in a real laboratory environment was not evaluated. We developed experiments with different feature vectors in order to select the best trade-off of computational time and performance. Finally, the Local Binary Patterns (LBP) [37]of its grey-level version, were used as inputs of the classifier. This feature vector was calculated for each cyst using the pixels inside its area, similarly to previous software generated by us [31,38]. Specifically, we used the LBP uniform patterns in order to develop a feature vector of suitable dimension to be used by the classifier. The histogram of the patterns’ labels accumulated over the cyst region was employed as texture descriptor. The feature vector was constructed concatenating the histograms of the LBP uniform patterns considering the following values for the radius R and neighbors P pixels (R,P) = {(1,8), (2, 16), (3, 24)}, developing a vector with 54 features [36,37].

### Cystic index, cyst number and size profile calculation

**Fig 2:**
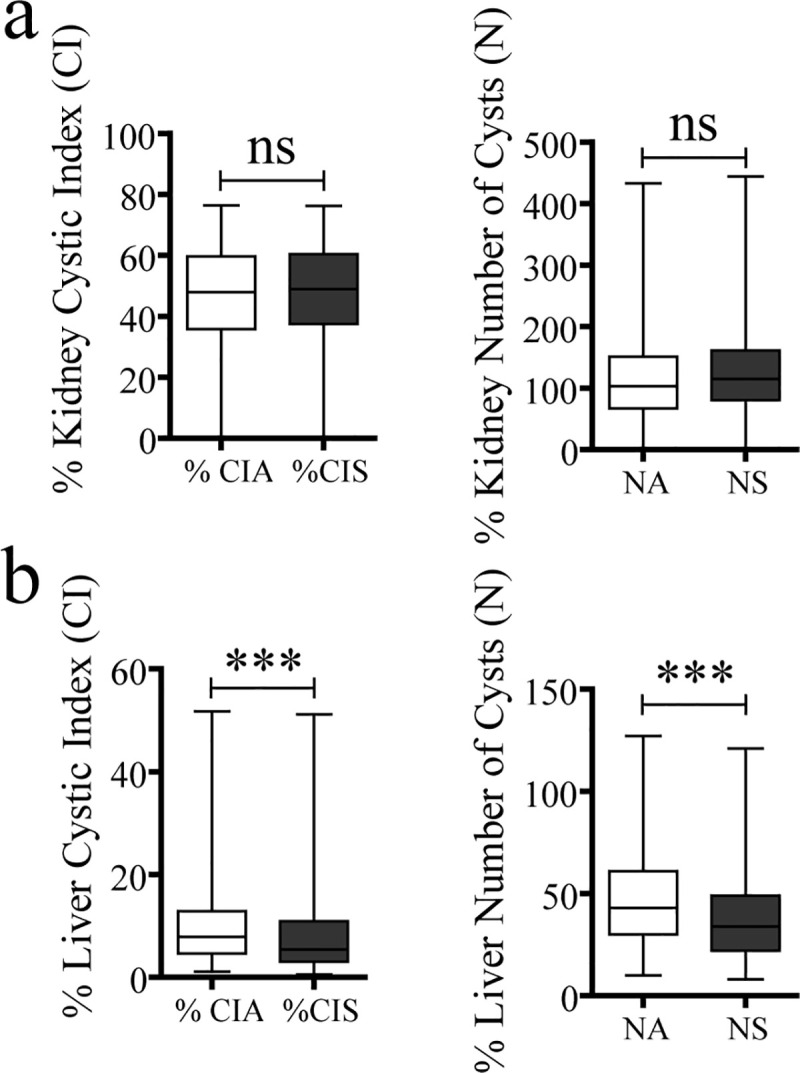
Cystic Index and Number of cysts analysis with supervised and non-supervised Automatic Cyst Recognition. Box plots show the comparison between Cystic Index Automated (CIA) versus Cystic Index Supervised (CIS) and between Number of cysts Automated (NA) versus Number of cysts Supervised (NS), respectively, for all kidney (a) and all liver (b) images. No significant differences were found between supervised and non-supervised Automatic Cyst Recognition in kidney images (a). However, supervise the Automatic Cyst Recognition significantly (*** p < 0,001) diminished the cystic index and number of cyst in liver samples (b). The lower and upper ends of the box define the 25% and 75% quantiles, respectively, the middle line in the box is the median of the values and the whiskers are the 0% and 100% quantiles. Mann-Whitney test was used for statistical analysis; ns: no significance; *** p < 0,001.

The *Automatic cyst recognition* and *Supervised post-processing* tools of CystAnalyser were tested in 464 liver and 331 kidney histological images. No differences were found between supervised and non-supervised Cystic Index (CIA versus CIS) and the number of cysts (NA and NS) in the kidney samples (Fig 2A). On the other hand, statistical significances were found in liver CI and liver N, indicating the necessity of supervision for the *Automatic cyst recognition* (Fig 2B).

**Fig 3:**
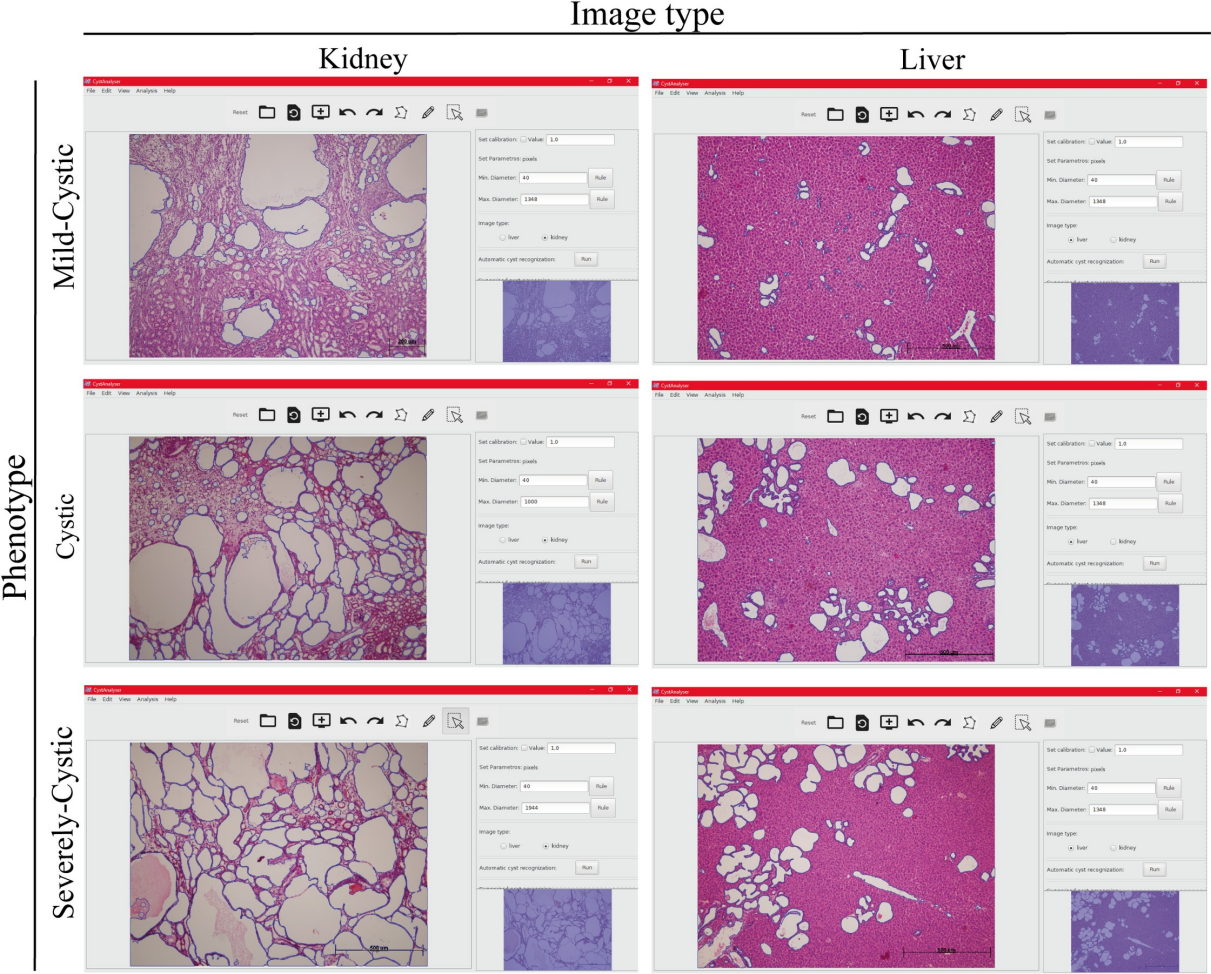
CystAnalyser Automatic Cyst Recognition in different group of severity of renal and hepatic cystic disease. Representative screenshots of CystAnalyser showing histological images of cystic liver and kidney after the application of the Automatic Cyst Recognition algorithms (cyst contour in blue). The left and right panels are respective histological images of kidney and liver. Upper panels show representative images of a mild-cystic phenotype, central panels are for cystic phenotype and bottom panels are for severely cystic phenotype.

**Fig 4:**
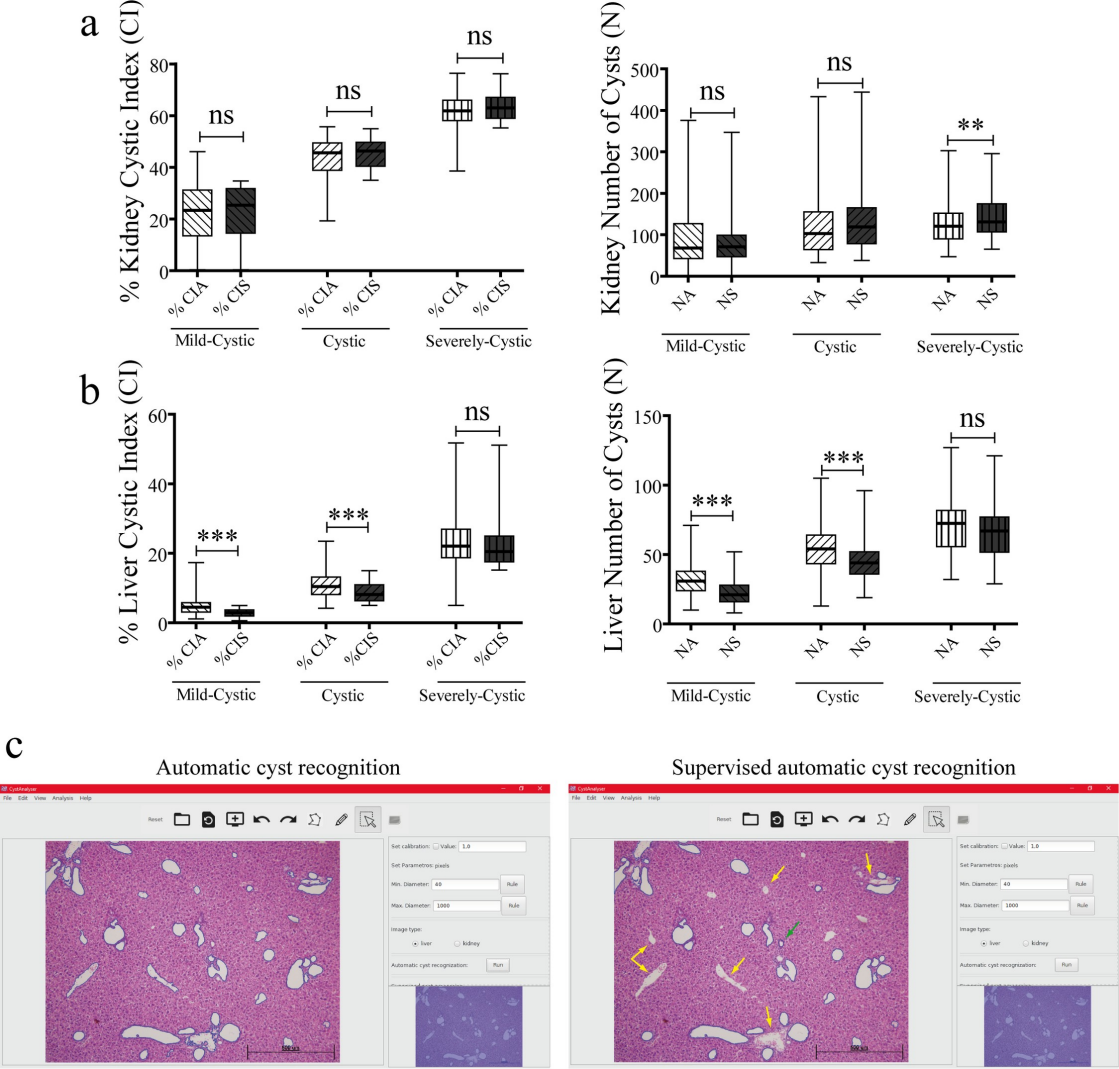
Comparison of supervised and non-supervised Automatic Cyst Recognition of CystAnalyser, according of cystic disease severity. (a-b) Box plots show the comparison between Cystic Index Automated (CIA) versus Cystic Index Supervised (CIS) and between Number of cysts Automated (NA) versus Number of cysts Supervised (NS), respectively, for kidney (a) and liver (b) images classified in mild-cystic, cystic and severely-Cystic. No significant differences were found between supervised and non-supervised Automatic Cyst Recognition in kidney images (a) with the exception of the number of cysts in the renal severely-cystic phenotype (** p < 0,01). Nevertheless, in hepatic mild-mystic and mystic images we observed significant differences (*** p < 0,001) in Cystic Index and the Number of cysts (b). The lower and upper ends of the box define the 25% and 75% quantiles, respectively, the middle line in the box is the median of the values and the whiskers are the 0% and 100% quantiles. Mann-Whitney test was used for statistical analysis; ns: no significance; ** p < 0,01; *** p < 0,001. (c) Representative example of mild-cystic liver with Automatic Cyst Recognition by CystAnalyser (left panel) and after Supervised Automatic Cyst Recognition (right panel). The yellow arrows show cysts erroneously recognized by the program or false positive, mostly bile ducts, hepatic vein and arteries. The green arrow shows a false negative or true cyst not detected by CystAnalyser. Bile ducts and hepatic vasculature represent an important part of hepatic cystic region recognized by CystAnalyser; which, when eliminated (with *Supervised post-processing*), implies a significant quantitative change in the cystic Index and number of cysts.

In order to identify possible inaccuracies due to the variable phenotype across samples, we established varied degrees of liver and kidney disease phenotypes (mild-cystic, cystic and severely-cystic) according to the CI (see the Materials and methods section). Fig 3 shows a representative example of the *Automatic cyst recognition* interface for the different degrees of disease severity in cystic kidney and liver histological samples. The *Automatic cyst recognition* interface tool was robust calculating kidney cystic Index and the number of cysts (Fig 4A), with a sensitivity (Se) and specificity (Sp) range of 76.9–78.6 and 77–89.7, respectively for all degrees of disease phenotype (Table 1A). Only in those scenarios where several big cysts become fused (severely-cystic phenotype) it was necessary to employ the Supervised *post-processing* tools by an expert user (Table 1A, Severely Cystic NA vs. NS p>0.01).

**Table 1:** Results of CystAnalyser Automatic Cyst Recognition. Summary of results of the Automatic Cyst Recognition of kidney (a, upper panel) and liver images (b, down panel). The table reports the average value of the following parameters. The four first columns show comparison between Cystic Index Automated (CIA) versus Cystic Index Supervised (CIS); columns 5 to 8 show the results of compare Number of cysts Automated (NA) a Number of cysts Supervised (NS); finally, columns 9 to 12 represent the value for: Percentage of Cysts Deleted (PCD) and Percentage of Cysts Added (PCA) by the user in the supervision, the Sensitivity (Se) and Specificity (Sp) calculated by Eq 1 (see Materials and methods section). Mann-Whitney test was used for statistical analysis; we considered a value p < 0.01 (**) or p < 0.001 (***) as significant and a value p>0.05 as non-significant (ns).

Group	CIA	CIS	p-value	Sig.	NA	NS	p-value	Sig.	PCD(%)	PCA(%)	Se(%)	Sp(%)
**A**	**Kidney**											
Mild Cystic	22.25	21.76	>0.05	ns	82.97	96.46	>0.05	ns	23.04	21.92	77.94	76.96
Cystic	45.66	43.98	>0.05	ns	131.0	120.6	>0.05	ns	12.38	23.17	76.84	87.62
Severely cystic	63.26	61.85	>0.05	ns	142.32	126.63	<0.01	**	10.34	21.40	78.59	89.66
**B**	**Liver**											
Mild Cystic	2.89	4.74	<0.001	***	22.79	32.30	<0.001	***	36.58	9.17	90.25	63.42
Cystic	8.90	10.98	<0.001	***	57.5	61.0	<0.001	***	20.63	12.38	87.29	79.38
Severely cystic	22.78	23.84	>0.05	ns	66.6	71.52	>0.05	ns	10.93	6.20	93.8	86.07

On the other hand, we observed that CI and N of mild-cystic and cystic livers needed to be calculated supervising the *Automatic cyst recognition*, using *Supervised post-processing* tools, due to non-specific recognition of extrahepatic bile ducts and vasculature structures (Fig 4B and Table 1). The sensitive range was 87.29–93.80 and the specificity range was 63.42–86.07, the lowest values corresponding to the mild-cystic phenotype (Table 1B). Fig 4C shows an example of *Automatic cyst recognition* of a mild-cystic liver phenotype before (left panel) and after (right panel) user input by deletion of the extrahepatic bile ducts, hepatic veins and arteries in order to calculate the exact liver CI and N. Although it was an inconvenience, this result offered value to the plasticity of CystAnalyser providing both the supervised and non-supervised tools according to the phenotype, organ and user needs.

As we can see in Table 1A, the Number of cysts after Supervision (NS) was higher for kidney images (except for mild-cystic images) than the Number of cysts Automated (NA). However, this did not occur for liver images because NS is lower than NA (Table 1B), owing to the necessity to delete hepatic vasculature and extrahepatic bile ducts (see Fig 4B and 4C). In relation to this, the percentage of cysts recognized automatically by CystAnalyser and deleted by the user was lower in kidney than in liver (in Table 1, column PCD). Nevertheless, the percentage of cysts added by the user was higher in kidney than liver (in Table 1, column PCA). This fact was also reported by sensitivity and specificity (in Table 1, columns Se and Sp respectively). The sensitivity was higher than 87.29% and 76.84% for all groups of liver and kidney respectively, and specificity range of 76.96–89.66% for kidney and 63.42–86.07% for liver images. These results of Sensitivity and Positive Predictive Value were very promising, taking into account the laborious way to count the recognition errors in this study (see subsection Statistical analysis, Materials and Methods), because when the expert used the Supervised post-processing tools, one action of the user on the GUI implied counting several errors (always more than two).

**Fig 5:**
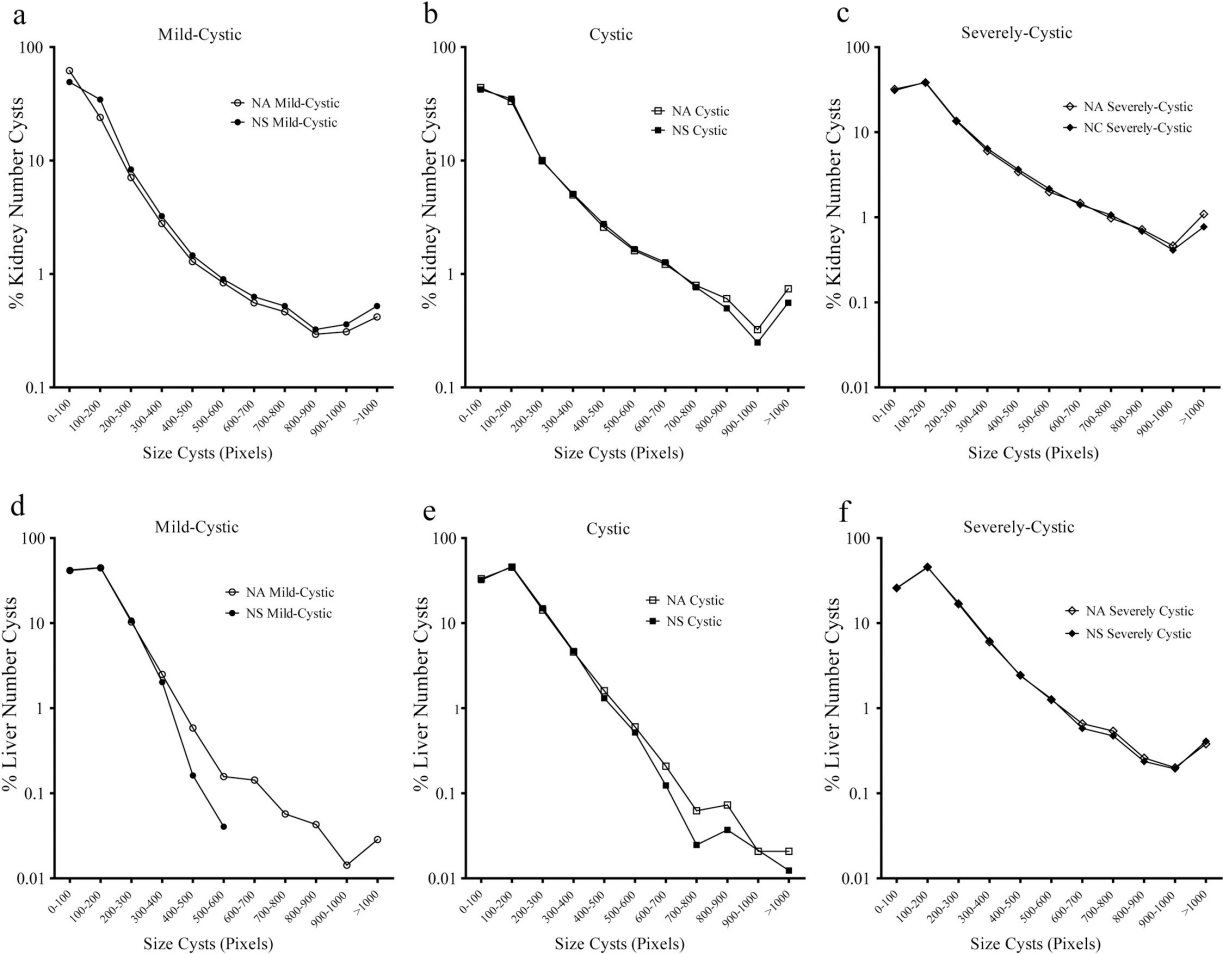
CystAnalyser allows studying the cystic profile, based on the size of the cysts. Logarithmic graph showing a profile of the cyst diameters automatically recognized by CystAnalyser (Number of cysts Automated, NA) versus the cysts reviewed (Number of cysts Supervised, NS) for kidney (a-c, upper row) and liver (d-f, lower row). In general, the cystic profiles were very similar except for the hepatic mild-cystic images (d); which after supervision of Automatic Cyst Recognition the biggest cysts disappeared due to deletion of bile ducts and hepatic vasculature (see Fig 4B and 4C). Kidney and liver images were classified in the different group according severity of cystic disease. The cyst size is shown in pixels.

In order to analyze the behavior of CystAnalyser with the cyst profile, the size or diameter of each individual cyst was preset. Fig 5 shows the Number of cysts Supervised (NS) and Cysts Automatically detected by CystAnalyser (NA) for all kidney (Fig 5A–5C) and liver (Fig 5D–5F) images featuring all the variable cystic phenotypes. In general, the cyst size profiles were quite similar. The main difference was the mild cystic images for the smaller cysts (diameter lower than 300 pixels) in the kidney (Fig 5A); specifically, for the cysts with diameters between 0 and 100 pixels the difference between NA vs NS is 12.01 points, and for the cysts with diameters between 100 and 200 pixels the difference was -10.56 points. The reason being the program had difficulty in recognizing the smallest renal cysts, with narrow lumens, which required marking or completing it manually. However, in the liver (lower panel) the main difference was for the bigger cysts (diameter higher than 500) in mildly cystic and cystic phenotypes, with a difference of 1.75 points for both between NA vs NS. As mentioned and shown in Fig 4C, the extrahepatic bile ducts must be deleted from the recognized cysts and this generated a difference in the cystic index and cyst size profile, as it occupied an important part of the image.

### Comparison with ImageJ

**Fig 6:**
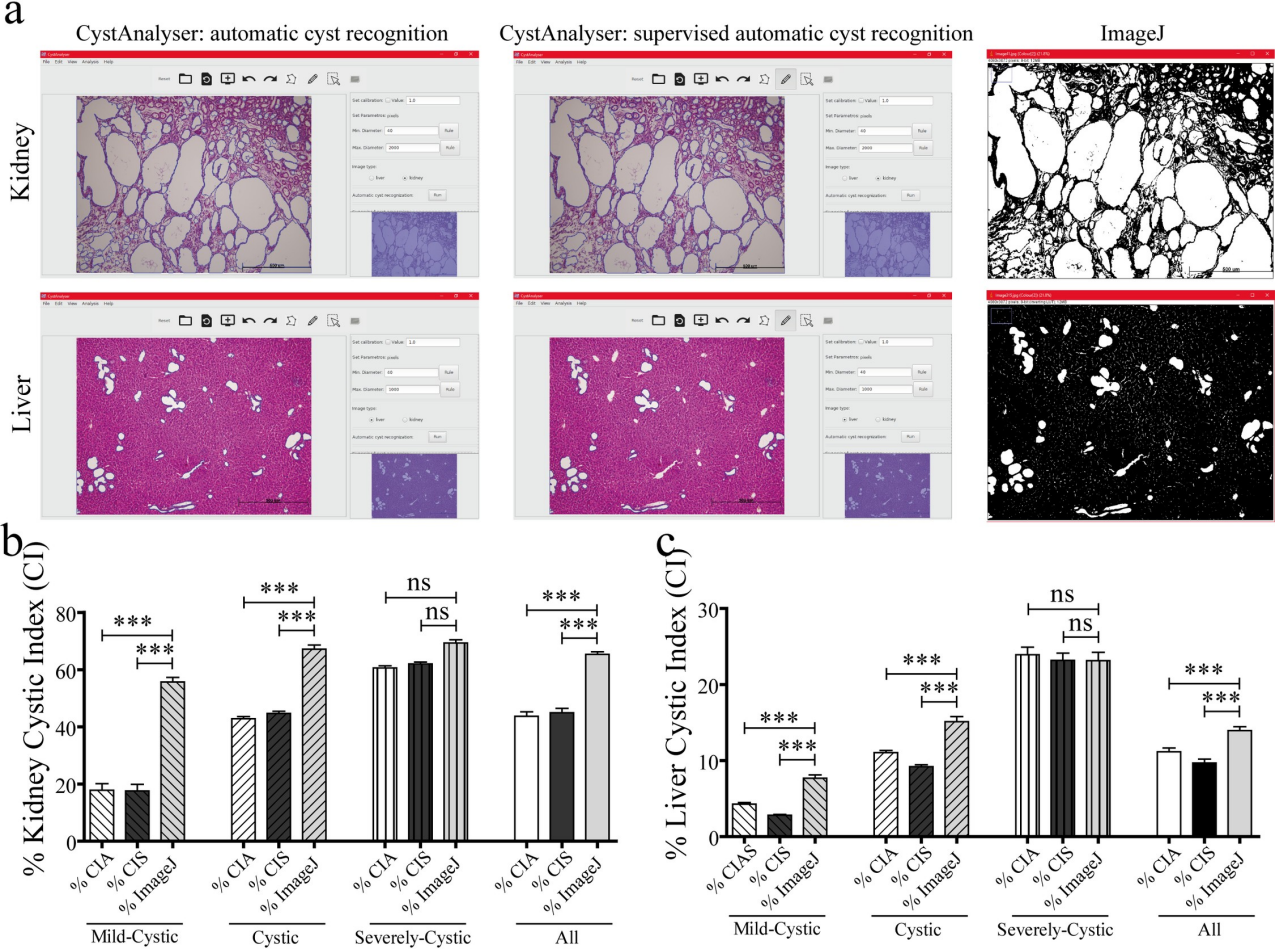
CystAnalyser provides a more reliable Cystic Index value than ImageJ. (a) Cyst recognition in a cystic kidney (upper panel) and liver (lower panel) images using CystAnalyser without supervision (left image), after user supervision (middle image) and using ImageJ. Cysts contours were overlapped to image for CystAnalyser (color blue); whereas for ImageJ the white regions were counted as cystic area. (b-c) Bar plots showing the cystic index of CystAnalyser (automated or supervised) versus Cystic Index provided by ImageJ for kidney (b) and liver (c). In general, we observed that CystAnalyser provided a more reliable and accurate Cystic Index than ImageJ; and in the same way, CystAnalyser discriminated better the different images according to severity of cystic disease. We found significant differences (*** p < 0,001) in Mild-Cystic and Cystic images (b-c). However, in severely-cystic images the cystic index provided by CystAnalyser and ImageJ were similar. CIA: Cystic Index Automated (provided by CystAnalyser). CIS: Cystic Index Supervised (provided by CystAnalyser). Bars represent mean + Standard Error (SE). Kruskal-Wallis test, with Dunn multiple pair-wise comparison post-test, was used for statistical analysis; ns: no significance; *** p < 0,001.

Next, we compared the cystic index calculated by CystAnalyser (automated or CIA, and supervised or CIS) with ImageJ, which has been the software most used for this analysis. For the comparison study, we analyzed 128 images of kidney; 29 mild-cystic, 52 cystic and 47 severely-cystic and 287 images of liver; 111 mild-cystic, 115 cystic and 61 severely-cystic. Examples of the visual results of cystic kidney and liver images provided by CystAnalyser (before and after user supervision) and by ImageJ are shown in Fig 6A. We can see that ImageJ recognizes white parts as cysts, predominantly between cysts or zones in the renal medulla (near to renal papilla) in the case of kidney images, and hepatic parenchyma in the case of liver images. Besides, given that ImageJ did not allow supervision after the automatic recognition of the cysts which increased likelihood of false positives (for example tubes and dilated tubes for kidney, extrahepatic bile ducts in the liver etc.) in the final result.

In order to perform a statistical comparison, Fig 6B and 6C show the cystic index calculated for all images, grouped by cystic phenotype for kidney (Fig 6B) and liver (Fig 6C), using: CystAnalyser without any user supervision (CIA), CystAnalyser after user supervision (CIS) and ImageJ. The difference between the cystic index calculated by CystAnalyser and ImageJ was significant for all cystic degrees except for the severely-cystic phenotype. ImageJ provided an overestimated result for both tissues, especially in kidney images since the difference between CIS (which is the most accurate value) and the cystic index of ImageJ was 38.07 points (mild-cystic), 22.45 points (cystic) and 7.28 points (severely-cystic). Consequently, CystAnalyser overcame ImageJ in precision. On the other hand, to do the quantitative analysis in ImageJ the segmentation threshold setting was required, a parameter which was implicit because it’s meaning was not clear for the user, and its optimal value must be selected by trial and error, being different for each image. So, the cystic index calculated by ImageJ was subjective because it depended on the threshold selected by the expert, which may vary among experts. On the contrary, CystAnalyser only needed to set the range of cyst diameters to be recognized. This parameter was explicit (clearly understandable by the expert) and descriptive (it can be set in micrometers or pixels), and it was the same for all images, so the results of CystAnalyser were less subjective (or not subjective at all) with respect to the user who did the analysis.

In addition, CystAnalyser achieved a score of 80.75 in the System Usability Scale (SUS) questionnaire, used to measure the subjective perception of the system, which means that this software is considered good to excellent. CystAnalyser is fast enough to work online on a general-purpose personal computer in any biomedical laboratory, capable of developing automatic cyst recognition of an image almost instantaneously (approximately 3 seconds) and faster than ImageJ and requiring an average of approximately three minutes for manual expert supervision (ImageJ does not allow it), which could be avoided in program applications where automatic recognition is already accurate.

## Discussion

This work proposes CystAnalyser as a new reliable and easy to use software tool to evaluate cystic pathologies, such as polycystic kidney disease (PKD) and polycystic liver disease (PLD). CystAnalyser combines the automatic recognition of the cysts in histological images with a friendly GUI which allows to supervise the recognition before quantification. CystAnalyser provides the cystic index (area of cysts within the total area of a tissue), the number of cysts and the cystic profile (based on individualized cystic size) of a histological image.

In order to test CystAnalyser, we compared the cystic index and the number of cysts provided by CystAnalyser with the automatic cyst recognition tool with or without user input. A collection of 795 images was quantified by CystAnalyser (Table 2, see Materials and methods section), obtaining significant differences in the images of the liver but not the kidney (Fig 2). In order to make a more precise characterization to identify the cause of these differences and evaluate the reliability of the program, we classified the images into three groups according to the severity of the cystic phenotype (mild-cystic, cystic, and severely cystic; see Fig 3 and the Materials and methods section). The main differences were identified in the processing of mild cystic and cystic hepatic images, where it was difficult to distinguish small cysts from the extrahepatic bile ducts and hepatic vasculature for a novice in the field (Fig 4). Therefore, in this scenario, the input of an expert was recommended since the software error in determining the cystic index in mild cystic and cystic liver images was 39.03% and 18.94%, respectively. On the other hand, in kidney images, in general, there were no differences between the recognition of automatic supervised and unsupervised cysts, except for the number of cysts within severe cystic phenotype (Fig 4A, Table 1). There were no significant differences between the cystic profiles according to the size of the cyst obtained with and without supervision for both tissues (Fig 5). Regarding the sensitivity and specificity of the software, the value ranges were 76.84–90.25% for the kidney and 63.4–89.66% for the liver, respectively (Table 1). These values weare intentionally underestimated, because the software used a strict methodology to over count errors (dividing a cyst into two different counts as several errors and not as one). For this reason, CystAnalyser allowed users to monitor or supervise the default quantification process to obtain results with greater precision and robustness.

**Table 2:** Number images used in the work. Number of kidney and liver images used in this work for each animal model and cystic phenotype, according the classification used. p: postnatal day of sacrifice.

Animal model	Group			
	Mild Cystic (n)	Cystic (n)	Severely Cystic (n)	Total (n)
	Kidney			
*Pkd1*^*cond/cond*^*;Tam-Cre* p30	67	126	33	226
p45	0	15	90	105
Total	67	141	123	331
	Liver			
*Pkd1*^*cond/cond*^*;Tam-Cre* p30	187	56	2	245
p45	26	65	11	102
*Pkhd1*^*del3-4/del3-4*^ p250	4	56	57	117
Total	217	177	70	464

With the aim of assessing the potential of this new software, we also compared CystAnalyser versus, the software most commonly used in scientific research, ImageJ. CystAnalyser was shown to outperform ImageJ in precision and reliability, being less subjective even without user supervision. In addition, CystAnalyser provided additional functionalities to calculate the number of cysts, the cystic profile based on their diameter and the possibility of monitoring and modifying the cysts identified by the software before quantification. The number of cysts and the cystic profile are necessary for the characterization of the phenotype and, therefore, the pathophysiology of the polycystic disease, complementing the cystic index, which is the most used biomarker in polycystic research (Manuscript under review).

Future work on this software will focused on the development of new functionalities in automated microscopes in order to perform an automatic quantitative analysis of serial images. Supervised machine learning techniques will also be used to avoid post-processing of recognized cysts by decreasing the number of false positives, increasing the specificity of automatically detected results, and reducing the time and effort of expert supervision. Similarly, we would like to extend this automatic detection application to other techniques such as immunofluorescence (in order to specifically mark and quantify cysts from different parts of the nephron) and to other fields of nephrology such as glomerulopathies (automated quantification of the number and type of glomeruli).

## Materials and methods

### Animal models

C57/BL6 *Pkd1*^*cond/cond*^*;Tam-Cre* and C57/BL6 *Pkhd1*^*del3−4/del3−4*^ were used in the present study [14–16]. In the *Pkd1*^*cond/cond*^*;Tam-Cre* model we induced Cre recombinase activity, in order to inactivate the *Pkd1* gene, in mice postnatal 10 and 11 days of age by intraperitoneally injecting nursing mothers with tamoxifen (10 mg/40 g) in corn oil (Sigma-Aldrich) [16]. Mice were sacrificed at postnatal day 30 (56 mice) or 45 (15 mice). The *Pkhd1*^*del3−4/del3−4*^ mice were sacrificed with more than 8 months of age (14 mice). We used a total number of 85 mice in this study, performed using protocols approved by the Universidade de Santiago de Compostela Animal Care and Bioethical Committee (#15010/2020/002) and the mice were house in pathogen-free facility (SPF).

### Immunohistochemistry and image acquisition

After mouse euthanasia, the kidneys and livers were washed in PBS 1X and fixed with 4% paraformaldehyde (pH 7.4) for at least 10 hours. Tissues were washed again with distilled water for 2 hours and rehydrated with 50% ethanol for 2h and finally kept in 70% ethanol at 4°C. The organs, once processed, were embedded in paraffin and sectioned at 4.5μm thickness. Sagittal kidney sections and liver cross-sections were stained for Hematoxylin-Eosin using a standard protocol, which were then visualized under an Olympus BX51 microscope connected to an Olympus Camera DP70 using a magnification of 10X. The number of images acquired from each tissue range from three to ten images, and their size is 4080 × 3072 pixels.

### Dataset description

The histological images of kidney and liver were grouped depending on their Cystic Index, according to value of CystAnalyser Cystic Index Supervised or CIS, in: “Mild-Cystic”, “Cystic” and “Severely-Cystic”. In kidney, we considered a “Mild-Cystic” when its cystic index was between 0 to 35%, “Cystic” between 35% to 55% and “Severely-Cystic” greater than 55%. For liver, we considered a “Mild-Cystic” liver when its cystic index was lower than 5%, “Cystic” between 5% to 15% and “Severely-Cystic” greater than 15%. Table 2 summarizes the number of images (795 in total) analyzed by CystAnalyser for each type of animal, Cystic Index and type of organ (kidney or liver).

### Statistical analysis

For the statistical evaluation of the automatic recognition algorithms, we considered that a cyst was correctly recognized, a true positive (TP) hit, if the user did not modify the outline provided by the recognition algorithm. A cyst was considered false positive (FP) whenever the user manually deleted the cyst or it was modified by some “tools for supervision”. A cyst was considered false negative (FN) whenever the user manually added one cyst or it was added by some “tools for supervision”. For example, when the user split a cyst, we counted it as one false positive (like deleted) and two false negatives (like added). If the user performed the operation *Remove inner cyst*, all the cysts inside a manually added cyst were considered as FP. If the user uses the *Complete* tool, we counted one FP and one FN. If the user performed a *Merge* operation, we counted it as FP the number of cysts merged and one FN. When the user clicks the button *Run* after the label *Add smaller cyst*, all the cysts added were considered as FP. Once we counted the TP, FP and FN for an image, we defined the sensitivity (Se) and specificity (Sp), both in %, as:

$$Se=\frac{100TP}{FN+TP}Sp=(1-\frac{FP}{NA})*100$$

Thus, the number of cysts in the image (after supervision) was NS = TP + FN and the number of cysts automatically recognized by the computer was NA = TP + FP.

The normality of the data was assessed using D’Agostino-Pearson, Kolmogorov-Smirnov and Shapiro-Wilk tests for all groups of data. Non-parametric methods were always required so, we calculated the statistical significance by Mann-Whitney non-parametric or Kruskal-Wallis non-parametric one-way ANOVA test with Dunn multiple pair-wise comparison posttest. We considered a value p < 0.05 as significant. The statistical analysis was performed using Prism version 7.0 for Windows (GraphPad software).

### Experts perception

The System Usability Scale (SUS) is a free questionnaire to measure the learn ability and subjectively perceived usability of computer systems [39,40]. This questionnaire has 10 items with five-point scale ranging from 1 (strongly disagree) to 5 (strongly agree). The final system score was provided in a scale from 0 to 100. The score was calculated as following: for items 1, 3, 5, 7 and 9 (the positively worded items) subtract one from the user responses and for items 2, 4, 6, 8 and 10 (the negatively worded items) subtract the user responses from five. This scales all values from 0 to 4 (being 4 the most positive response). The converted responses were added for each user and multiplied the total by 2.5 to convert the scale from 0 to 100 instead of from 0 to 40. The SUS score is translated to people’s rating of systems and products in terms of adjectives in order to give a meaning to the SUS scores [41]. The criteria used was: SUS score below to 25 was a system worst imaginable; from 25 to 39 was from worst imaginable to poor; from 39 to 52 was from poor to ok; from 52 to 73 was ok to good; from 73 to 85 was good to excellent and from 85 to 100 was excellent to best imaginable. Thus, a small sample (8–12 users) was enough to give a good assessment of how people see your system or product. CystAnalyser was evaluated using SUS questionnaire by 10 researchers of Nephrology Lab with different expertise using it. The mean score achieved was 80.75, which means that CystAnalyser is from good to excellent. It is important to emphasize that half of the researchers scored CystAnalyser with more than 85 points, so they think that the software is from excellent to best imaginable.

## Supporting information

S1 Text CystAnalyser user guide. A detailed user guide about CystAnalyser is provided in order to facilitate the use of the program and its several functions.
(PDF)
