# Supplementary material for: CystAnalyser: A new software tool for the automatic detection and quantification of cysts in Polycystic Kidney and Liver Disease, and other cystic disorders
Source: PLoS Comput Biol. 2020 Oct 22;16(10):e1008337. doi: 10.1371/journal.pcbi.1008337 (PMC7608985; doi:10.1371/journal.pcbi.1008337)
Supplement: S1 Text — A detailed user guide about CystAnalyser is provided in order to facilitate the use of the program and its several functions. (PDF) [file pcbi.1008337.s001.pdf]

S1 Text
CystAnalyser User Guide 2020

October 13, 2020

Contents

1 What is CystAnalyser? 1

2 Installing CystAnalyser in Operating System Windows 2

3 Installing and running CystAnalyser in Operating System Linux 4

4 Run CystAnalyser in Operating System Windows 5

5 Set preferences 7
- 5.1 Calibration . . . 9
- 5.2 Diameters . . . 9
- 5.3 Work directories . . . 9
- 5.4 Draw preferences . . . 9
- 5.5 Set or change the configuration . . . 10

6 File menu 11

7 Edit menu 14

8 View menu or lateral panel 15
- 8.1 Graphical tools to supervise the cyst recognition. . . . 18
- 8.2 Visualization of results. . . . 22

9 Menu Analysis 24

10 Menu Help 28

1 What is CystAnalyser?

CystAnalyser is a free software tool for measure and count the cysts of histological images of cystic liver and cystic kidney in the Polycystic Kidney Disease

(PKD) and Polycystic Liver Disease (PLD) fields. CystAnalyser is based on automatic digital analysis, also it provides a Graphical User Interface (GUI) to review the cyst recognition (if the user considers it appropriate) and provides a more reliable result. It is multiplatform software written is C/C++ programming language. That program allows obtaining the Cystic Index, which refers to the cumulative area of cysts within the total area of the kidney or liver, the number of cysts and a profile of cysts according size. CystAnalyser was developed in a scientific collaboration by the research groups: Nephrology Lab of the Instituto de Investigación Sanitaria de Santiago de Compostela (IDIS)[1] and Centro de Investigación en Tecnoloxías da Información (CITIUS)[2] of University of Santiago de Compostela. The present user manual is organized as follows: section 2 describes the installation steps; section 4 describes how to run the Graphical User Interface (GUI) of CystAnalyser. Section 5 describes the configuracion of the work preferences in CystAnalyser. Sections 6, 7 and 8 label File, Edit and View menus, respectively. In Section 9 you can find information about how analyses the results obtained with CystAnalyser. Finally, Section 35 refers to Menu Help.

2 Installing CystAnalyser in Operating System Windows

The installation process is the common process to install programs. First, do double click on the file `setupCystAnalyzer.exe`. Then, it is open a window asking us about to allow to install a foreing program `setupCystAnalyzer.exe` on your computer. After clicking "Yes", the window of Figure 1 is open. If you click "Next", the program go on intalling.

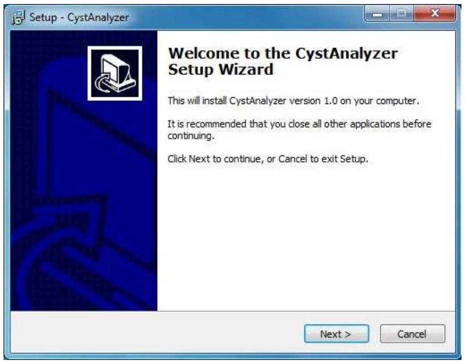

Figure 1: Window to install CystAnalyser.

The next intallation window is shown in Figure 2 to choose the intallation

[1] `http://www.idisantiago.es/`
[2] `http://citius.usc.es/`

folder. By default, this directory is CystAnalyzer (we recomend do not change the directory). Afterwards, click the button "Next" to go to the window shown in Figure 3.

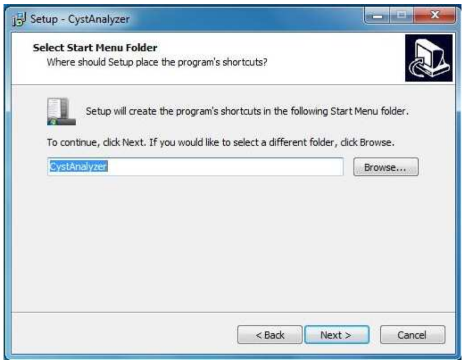

Figure 2: Window to install CystAnalyser. Select start menu folder.

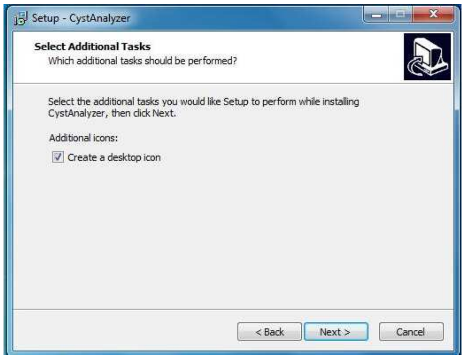

Figure 3: Window to install CystAnalyser. Select additional tasks.

Figure 3 asks if you want to create a desktop icon in the desktop. We recomend you activate the check box because it will be faster the access to CystAnalyser. Then, you click on the button "Next" to go to the next window (Figure 4). It is a window to confirm if you want to install CystAnalyser (clicking with the mouse on button "Install") or cancel the installation (clicking the button "Cancel").

After starting the intallation process, it will pop up a dialog showing the intallation process, as it can be seen in the Figure 5. This process can take a few seconds. If you click on the button "Cancel", the installation process will be cancelled. When the intallation process finished, it will be shown the window of Figure 6. In this window, you click the "Finish" button to finish the installation process. If the check box *Lunch CystAnalyser* is marked, CystAnalyser will be run. Otherwise, CystAnalyser will not run now, but you can run every time

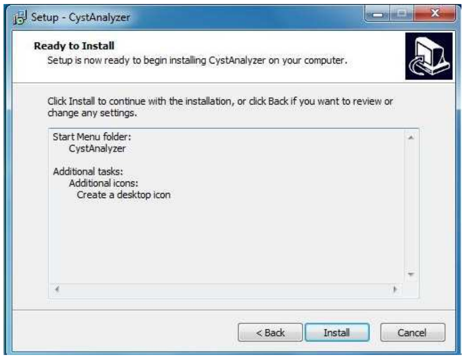

Figure 4: Window to install CystAnalyser. Ready to install CystAnalyser.

lated double clicking the desktop icon of CystAnalyser, whick was created in the desktop during the installation process.

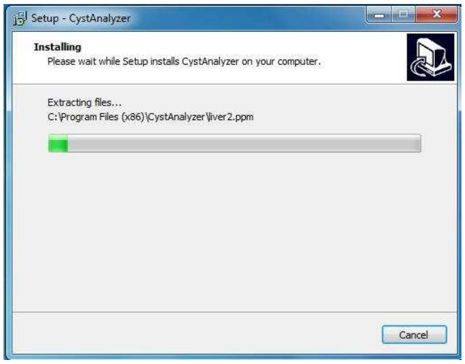

Figure 5: Window to install CystAnalyser. Installing CystAnalyser.

3 Installing and running CystAnalyser in Operating System Linux

In Linux, there are several types of packages, and every distribution has its own preferred package format. Ubuntu distributions used the Debian packages (format DEB). The .deb/Debian files containing CystAnalyser is the file `cystanalyser_1.0_all.deb`. It is provided the linux package for Ubuntu 18.04 version.

To install CystAanlyser, go to terminal, change to the folder where the file `cystanalyser_1.0_all.deb` is and type the following command:

```
sudo dpkg -i cystanalyser_1.0_all.deb
```

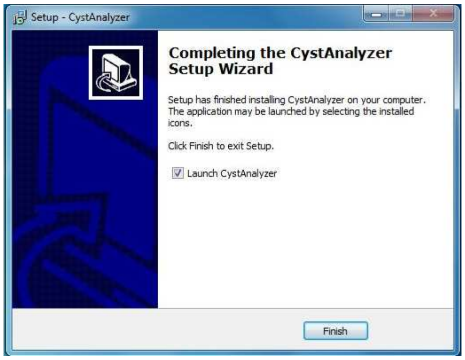

Figure 6: Window to install CystAnalyser. Completing the CystAnalyzer Setup Wizard.

and the system asks you by the administrator password.

You need to add dependencies typing the following command:

```
apt-get install -f
```

After typing the above command, dependencies will be added to your machine and your Debian package (.deb) file will be installed. Then, you can run CystAnalyser using the following command:

```
./cystanalyser
```

CystAnalyser can be removed from the computer using the command:

```
sudo apt remove cystanalyser
```

4 Run CystAnalyser in Operating System Windows

After installing CystAnalyser in your computer, doble click on the desktop icon of CystAnalyser to run it and the main window of Figure 7 will be open. It encloses a menu bar (at the top of screen) containing all menu commands, a toolbar (under the menu bar) containing tools to access to the main funcionality of CystAnalyser, and an image window (at the bottom of screen) in which the histological images are open. The menu bar lists all **CystAnalyser** commands. It is organized in five menus:

1. **File**: Basic file operations (opening images and XML files, saving cysts data in files and statistical results); set and load the work preferences; and exit of CystAnalyser.

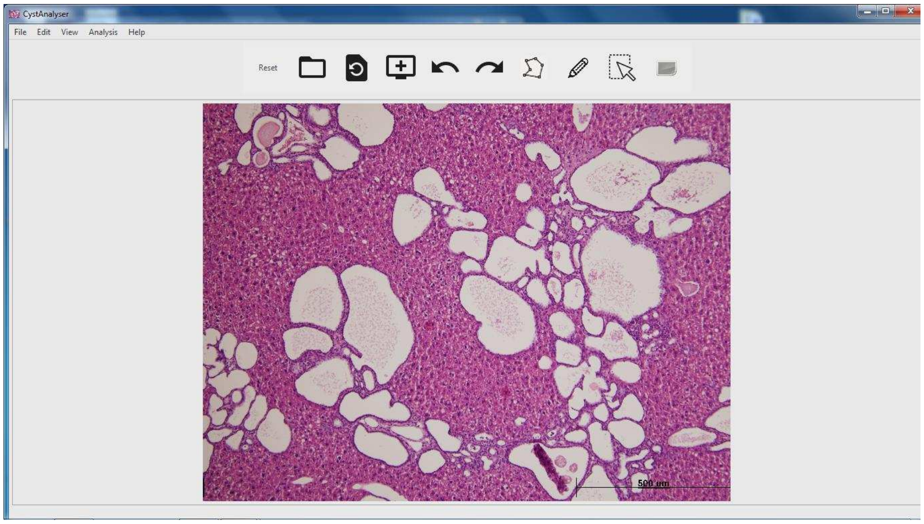

Figure 7: Main window of CystAnalyser with a histological image of cystic liver.

2. **Edit**: redo and undo operations, fit the image to the window size and set the image to the original size.

3. **View**: visibilize or hide the processing panel.

4. **Analysis**: provides functionalities to calculate the results, as Cystic Index, number of cysts and a histogram with the profile of cysts according to size (pixels or micrometers) to many images jointly.

5. **Help**: the help funcionality is not complete.

The toolbar is a fast access to the main funcionality of CystAnalyser, which contains the following icons (if the mouse is put on the icon, a pup up message shown the funcionality of the icon is open):

1. **Reset**: (first icon) clear all the objects drawn on the image.

2. **Open** : (second icon) open a dialog to select the histological image to be open (see section 6).

3. **Zoom Fit**: (third icon) fit the image zoom to the image window.

4. **Original Zoom**: (fourth icon) set the original image zoom.

5. **Undo**: (fiveth icon) when you click this button, undo the last cyst drawn on the image.

6. **Redo**: (sixth icon) when you click this button, redo the last cyst deleted.

7. **Draw with point**: (seventh icon) activate the draw of regions (in our case cyst) tool. While this button is pressed, the user draws a cyst marking points with the left button of the mouse and finishs the outline of the cyst when the user click the midle button of the mouse.

8. **Draw freehand**: (eighth icon) activate the draw freehand regions tool. When this button is pressed, the user can draw a freehand region pressing the left button of mouse and keeping it pressed while you are drawing the region. When you release the mouse button, the region is finished.

9. **Select**: (nineth icon) when this button is activate, you can select an object drawn on the image. The object is selected clicking into the object with the left button of the mouse. To select more than one object, keep the key **Ctrl** or key "Control" while selecting objects.

10. **Lateral panel**: (ten icon) open the lateral panel, which will be used to process and analyse the open image (see the section 8 and Figure 8).

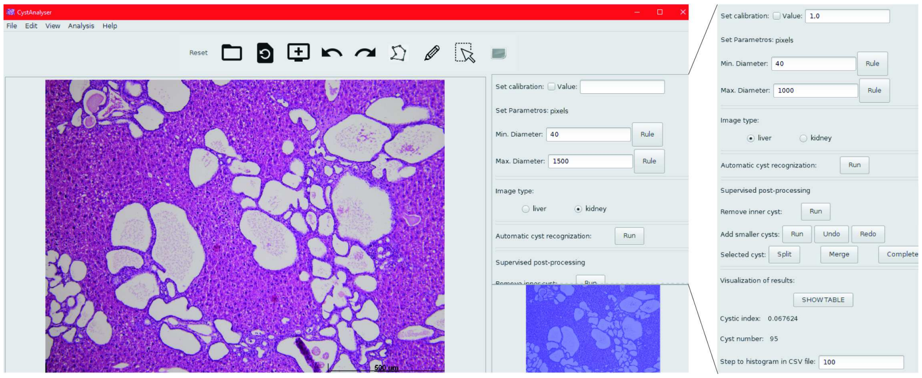

Figure 8: Main window of CystAnalyser with a typical histological image of cystic liver and the lateral panel open.

In the following sections, the funcionality of the Graphical User Interface (GUI) of CystAnalyser will be described. Section 5 describes the configuracion of the work preferences in CystAnalyser.

5 Set preferences

The submenus of the File menu from the menu bar is shown in the Figure 9. As you can see in this Figure, you can set the preferences (submenus preferences), reset the default preferences if the preferences have been set in a previous sesion, and load the preferences from an external XML file.

Selecting the submenu "Preferences" of menu File, the window shown in Figure 10 (left panel) will be open. The items to configure are:

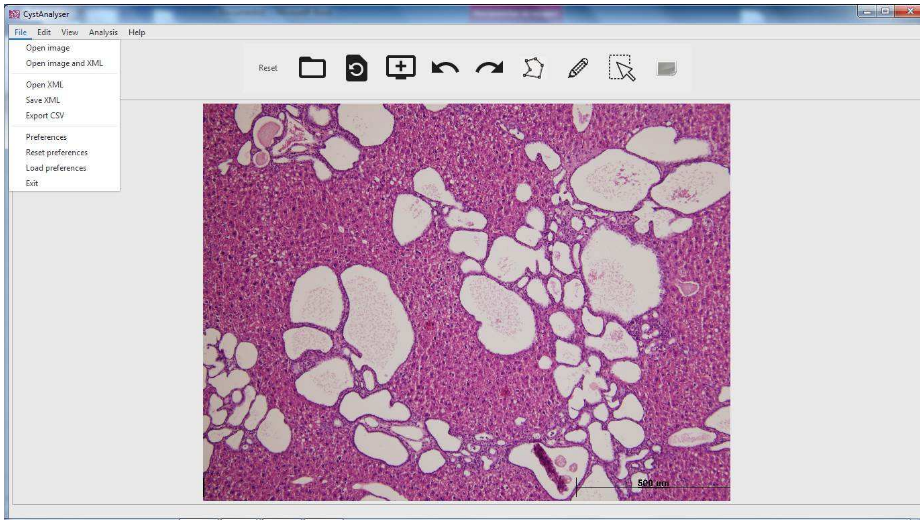

Figure 9: The option of menu to go *Preferences* option.

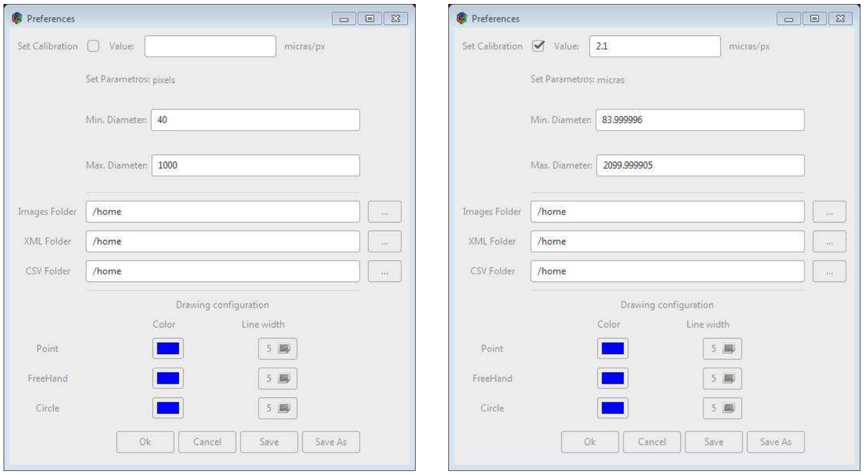

Figure 10: Window to configure the preferences of CystAnalyser: default configuration (left panel) and after configuration of calibration preference (right panel).

1. **Calibration**: it is the number of micrometers per pixel in the image. it allows to set the units to measure the objects (cysts in our case).

2. **Diameters**: it allows to put the minimum and maximum size of the cysts in the images. These parameters can be set in pixels (if the calibration is not set) or micras (if the calibration is set).

3. **Work directories**: it allows to set the default directories of the images, XML files and CSV files to use CystAnalyser.

4. **Draw preferences**: it allows to set the colour and line width preferences to shown the cysts overlapped to the images.

5. **Set or change the configuracion**: the buttons **Ok**, **Cancel**, **Save** and **Save As** at the bottom of Figure 10 set, cancel or save the configuration.

5.1 Calibration

The top of Figure 10 shown a check box after the label *Set Calibration.* This check mark assures that the Calibration real value has been added, if it is non-checked, then the calibration is considered in pixels. After the label *Value*, and if the check box of calibration is marked, you can set the number of micrometers per pixel in the image (press the "Enter" key after put the value). The calibration value are fixed by the digitalization process, depending on the magnification used in the microscope and the spatial resolution of the digital camera connected to it. The right panel of Figure 10 shows the left panel after activating the calibration and put its value to 2.1 micras per pixel. As it can be seen comparing both panels in Figure 10, when the check box of calibration is modified, the units and values of diameters change.

5.2 Diameters

CystAnalyser provides more versatility allowing the user to choose the minimum and maximum diameters of the cysts to recognize in the images. These parameters can be set after the labels *Min. Diameter* and *Max. Diameter* respectively, puting the values of minimum and maximum diameter (in micras if the calibration is active and in pixels if the check box of calibration is not marked). After set the diameters, you must press the "Intro" key to update the value in CystAnalyser.

5.3 Work directories

After the labels *Images Folder*, *XML Folder* and *CSV Folder* in Figure 10, there are their correspondent entry to visualize the default directory to store the images, XML files and CSV files respectively. After each entry there is a button with three points. Clicking this button open the file chooser dialog of Figure 11 to choose the work directory for images, XML files and CSV files respectively (Figure 12 shown the preferences window after modifying the work directories).

5.4 Draw preferences

After selecting the work preferences in Figure 12, you can change the colour and line width to draw the cysts overlapped to the image. Clicking with the mouse the blue button after the label *FreeHand* opens the colour chooser dialog

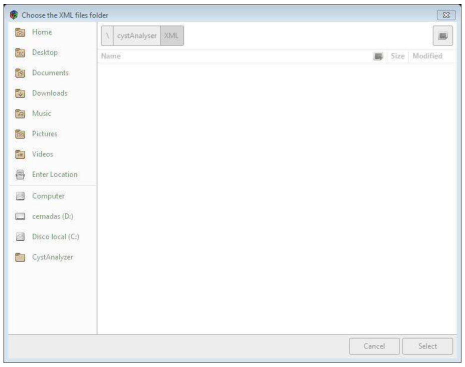

Figure 11: Window to choose a work directory.

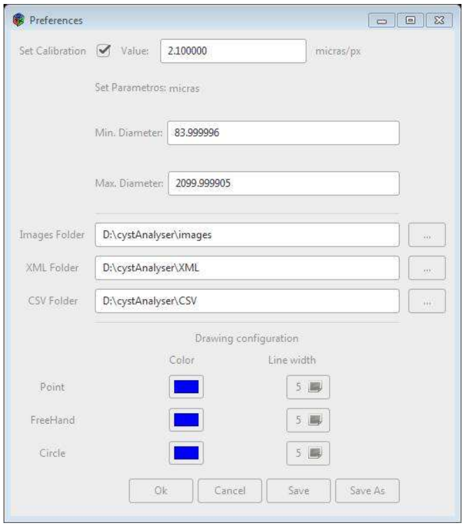

Figure 12: Window of preferences after setting the work directories.

shown in Figure 13. Selecting a colour in this window and pressing the button "Select", you change the drawing colour to freehand objects (cysts). Clicking in the button after the colour button, a drop-down list is open to select the line width to the freehand object.

5.5 Set or change the configuration

The four buttons **Ok**, **Cancel**, **Save** and **Save As** at the bottom of Figure 10 have the following funcionalities:

1. Clicking the button **Ok**, you set these preferences to the present work session.

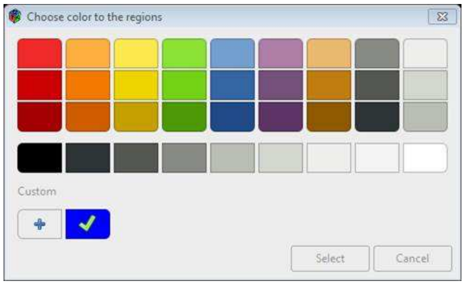

Figure 13: Window to choose the colour to draw cysts on the image.

2. Clicking the button **Cancel**, you cancel the operation of setting the preferences and the preferences will not be set.

3. Clicking the button **Save**, you set these preferences to the present work session and to another work session in the future.

4. Clicking the button **Save As**, you set these preferences to the present work session and CystAnalyser allows to store these preferences in a XML file, which can be loaded every time using the submenu "Load preferences" of menu File.

6 File menu

The **File** menu is within the menu bar (see Figure 9). The items available within this menu are: **Open Image**, **Open Image and XML**, **Open XML**, **Save XML**, **Export CSV**, **Preferences**, **Reset preferences**, **Load Preferences** and **Exit**. The **Exit** menu or the **X** button in the top-right side of window quit CystAnalyser. CystAnalyser work with three types of files: image files, XML (eXtensible Markup Language) and CSV (Comma-Separated Values) files. The image formats supported are the most frequently used like GIF, TIF, PNG, BMP, PPM, JPG, etc. There is one XML file per image, which saves the contours of the cysts, in order to allow that this analysis can be loaded into the program in other intant of time. The CVS file exports the statistical information of the quantitave analysis of the image.

The **Open Image** submenu or the second button of the toolbar opens the file chooser dialog of Figure 14. The directory open is the one set in the configuration of the preferences (see the section 5). Choose the path to the image and click the button **Open** in the bottom of the window to load the image in CystAnalyser. Take care that the image path is not biggest than 256 characters or the image path contains rare symbols, because Cystanalyser could not work correctly.

Figure 15 shows an image loaded in CystAnalyser with the cysts outline overlapped. When an image is loaded, the lateral panel is open and in the

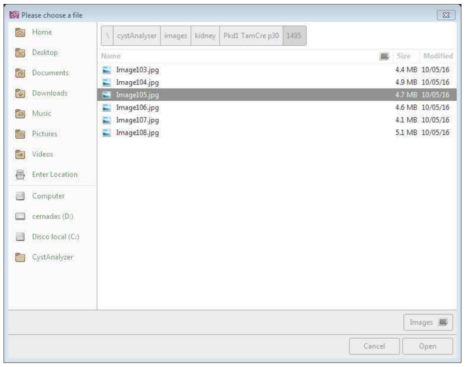

Figure 14: File chooser dialog to choose the image to be loaded in CystAnalyser.

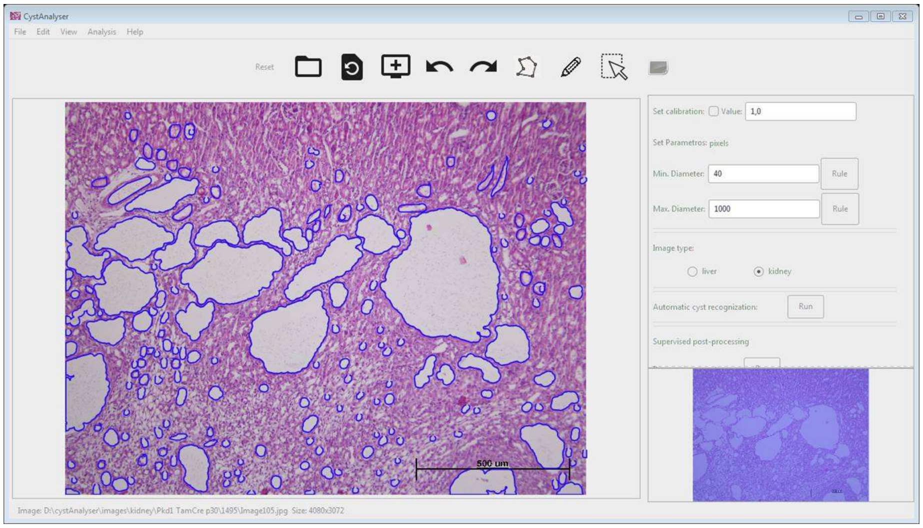

Figure 15: The image selected in Figure 14 is loaded in CystAnalyser.

bottom of the main window appear a state bar with the name of image file and its size. If the image was analysed in other intant and the XML file was stored, you can load this XML file and overlapp to the image selecting the submenu **Open XML**. This operation opens a file chooser dialog to choose the XML file (see the Figure 16) and overlapp the cysts contours to the image, as can be seen in Figure 15. The user must select the right XML file for each image, CystAnalyser does not check if this XML file corresponds to a specific image file.

The above process can be done in one step with the submenu **Open Image and XML**, which open a file chooser dialog, as in Figure 14, to choose the image to load and CystAnalyser checks in the XML path set in the preferences if there

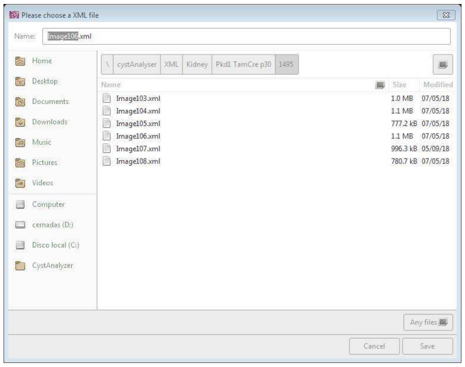

Figure 16: File chooser dialog to choose the XML file to be loaded in CystAnalyser.

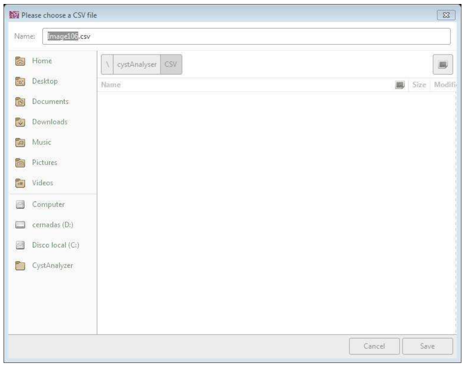

Figure 17: File chooser dialog to choose the CSV file to store the statistical analysis of the image.

is a XML file with the same name and extension xml. In this case, CystAnalyser opens this XML file and overlapps its content over the image loaded.

Once the cysts contours have been drawn on the image (automatically or manually), the submenu **Export CSV** opens the file chooser dialog of Figure 17. You set a file name (by default it is used the name of image with the extension CSV) and the program stores the statistical analysis of the image in that file and the CSV directory selected in the preferences. The information stored in the CSV file are: image path, number of cysts, calibration used, the diameter and area of all cysts (by default in pixels and in micras if the calibration is active), the cystic index and, finally, the profile of the cysts in the image (number of cysts in each size interval). The information of the CSV file can be loaded in a spreadsheet as LibreOffice Calc[3], as it can be seen in Figure 18.

[3] `https://www.libreoffice.org/discover/calc/`

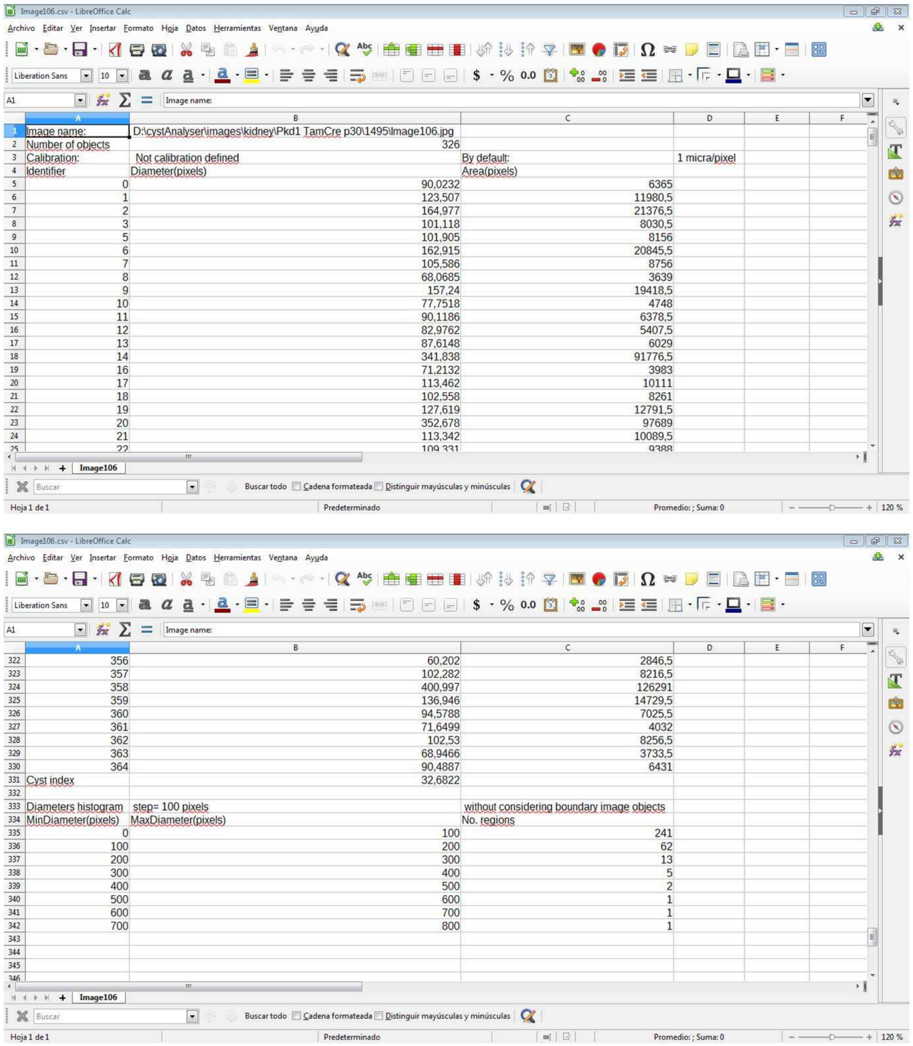

Row	A	B	C	D
1	Image name:	D:\cystAnalyser\images\kidney\Pkd1 TamCre p30\1495\Image106.jpg		
2	Number of objects	326		
3	Calibration:	Not calibration defined	By default:	1 micra/pixel
4	Identifier	Diameter(pixels)	Area(pixels)	
5	0	90,0232	6365	
6	1	123,507	11980,5	
7	2	164,977	21376,5	
8	3	101,118	8030,5	
9	5	101,905	8156	
10	6	162,915	20845,5	
11	7	105,586	8756	
12	8	68,0685	3639	
13	9	157,24	19418,5	
14	10	77,7518	4748	
15	11	90,1186	6378,5	
16	12	82,9762	5407,5	
17	13	87,6148	6029	
18	14	341,838	91776,5	
19	16	71,2132	3983	
20	17	113,462	10111	
21	18	102,558	8261	
22	19	127,619	12791,5	
23	20	352,678	97689	
24	21	113,342	10089,5	

Row	A	B	C
322	356	60,202	2846,5
323	357	102,282	8216,5
324	358	400,997	126291
325	359	136,946	14729,5
326	360	94,5788	7025,5
327	361	71,6499	4032
328	362	102,53	8256,5
329	363	68,9466	3733,5
330	364	90,4887	6431
331	Cyst index	32,6822	
332			
333	Diameters histogram	step= 100 pixels	without considering boundary image objects
334	MinDiameter(pixels)	MaxDiameter(pixels)	No. regions
335	0	100	241
336	100	200	62
337	200	300	13
338	300	400	5
339	400	500	2
340	500	600	1
341	600	700	1
342	700	800	1

Figure 18: An example of CSV file of the image of Figure 15 imported in LibreOffice Calc. The upper panel is the beginning of the file and the bottom panel is the end of the file.

7 Edit menu

Figure 19 shows the content of **Edit** menu, which is in the menu bar. The items available within this menu are also available in the toolbar (see section 4). They are: **Undo** (fiveth icon in the toolbar), **Redo** (sixth icon in the toolbar), **Fit Image** (third icon in the toolbar) and **Original Size** (fourth icon in the

toolbar).

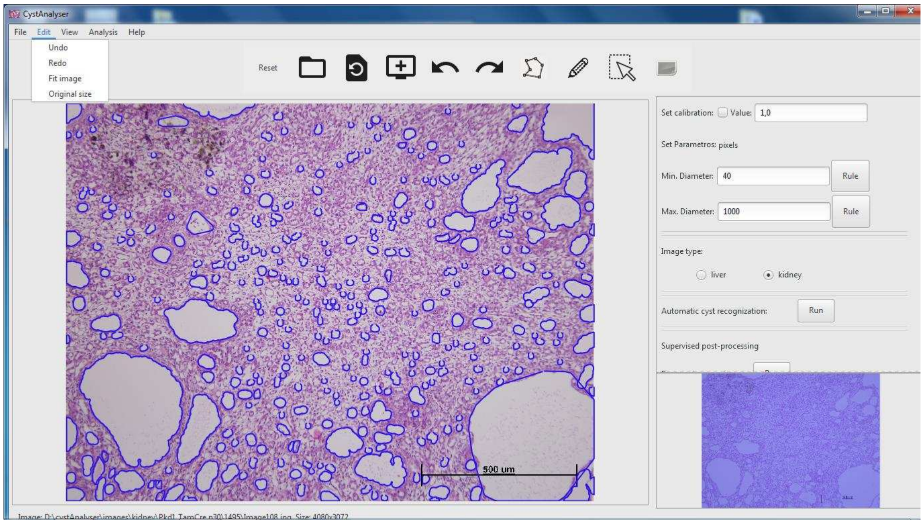

Figure 19: Funcionality available in the Edit menu of CystAnalyser.

The **Fit Image** item fits the image zoom to the image window and the **Original Size** sets the original image zoom. Another image zoom can be achieved rolling up the mouse wheel to increasse the zoom and rolling down the mouse wheel to decreasse the zoom. If the lateral panel is open, the position of the visible image area in the image window can be seen in the icon image located in the bottom of lateral panel (see Figure 20). Keeping this zoom, you can move to another part of the image by two methods: 1) pressing simultaneously the right buttons of the mouse and move the mouse to displace the visible area; and 2) press the left mouse button on the blue square in the icon image of the lateral panel and move it. Both the visible area in the window image and the icon image of lateral panel are synchronized. As can be seen, the overlays of the image are zoomed with the image.

8 View menu or lateral panel

Figure 21 shows the content of **View** menu in the menu bar. The only item available is **Processing Panel** (also included in the last icon of the toolbar), which closes or opens the lateral panel of figures 21 and 8.

The lateral panel contains the following funcionality from top to bottom:

1. **Calibration** (first line): shows the calibrations preferences set in the program or allows to change the calibration options as in the configuration preferences window (see section 5).

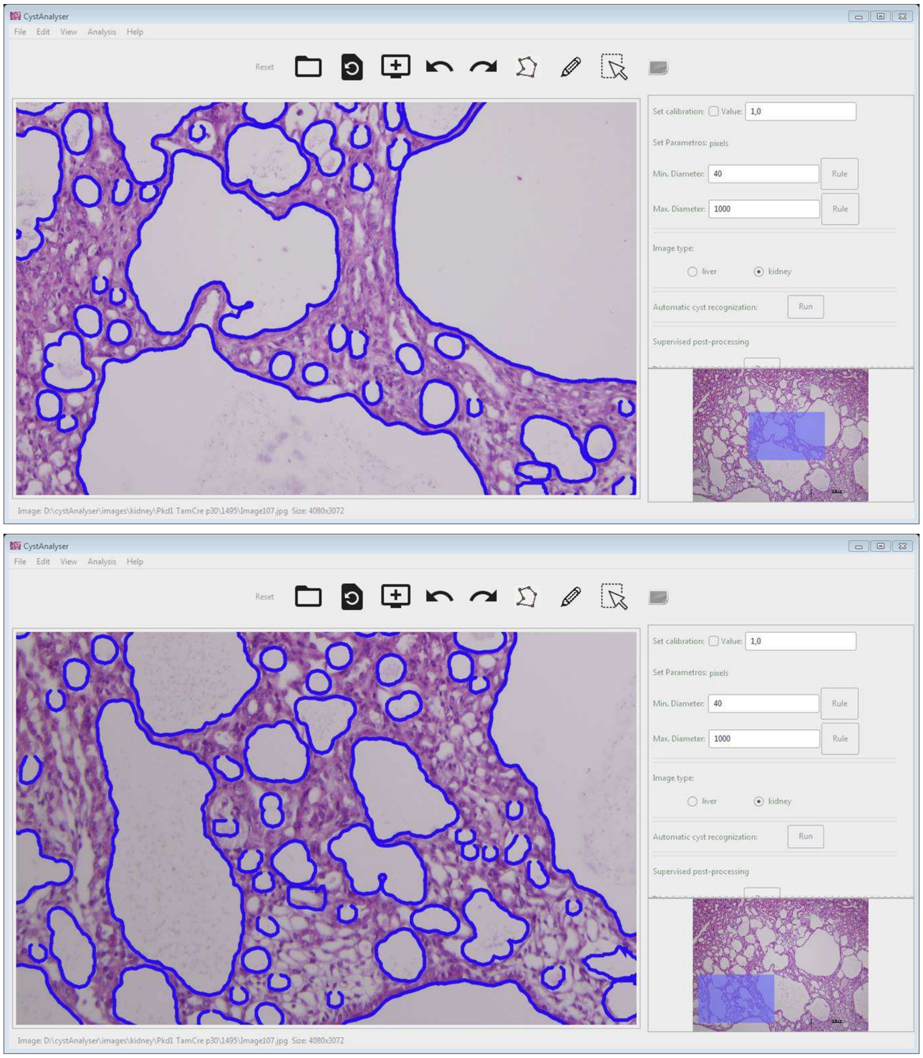

Figure 20: A zoom of the image in CystAnalyser.

2. **Diameters** (third and fourth line): shows the minimum and maximum diameters set in the preferences of the program or allows to set these values. The values of the minimum or maximum diameter can be set numerically, using the entry widget (as in the preferences window), or graphically, using the button **Rule**. When the calibration or diameters are modified, all the overlays of the image are deleted. Using the entry widgets, the value of the diameter would be provided in pixels, if calibration is not activated, or in micrometers otherwise. To choose the diameter graphically,

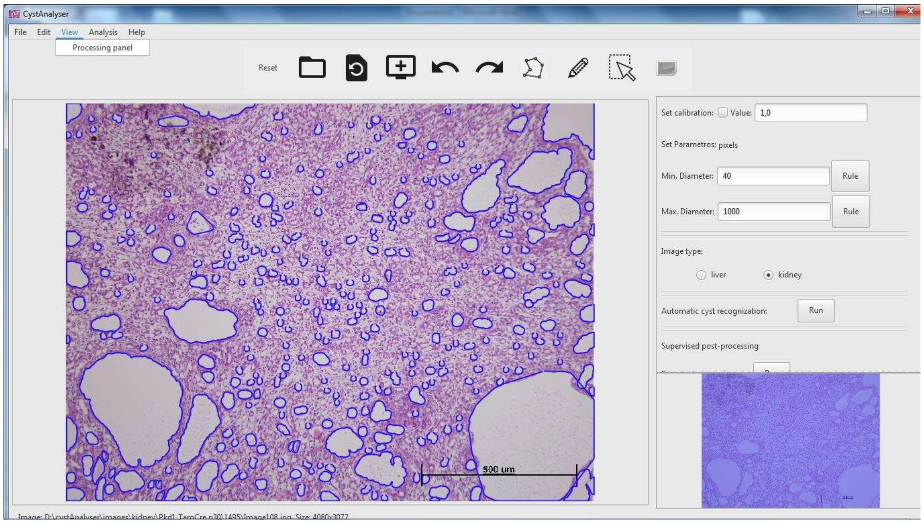

Figure 21: Funcionality available in the **View** menu of CystAnalyser.

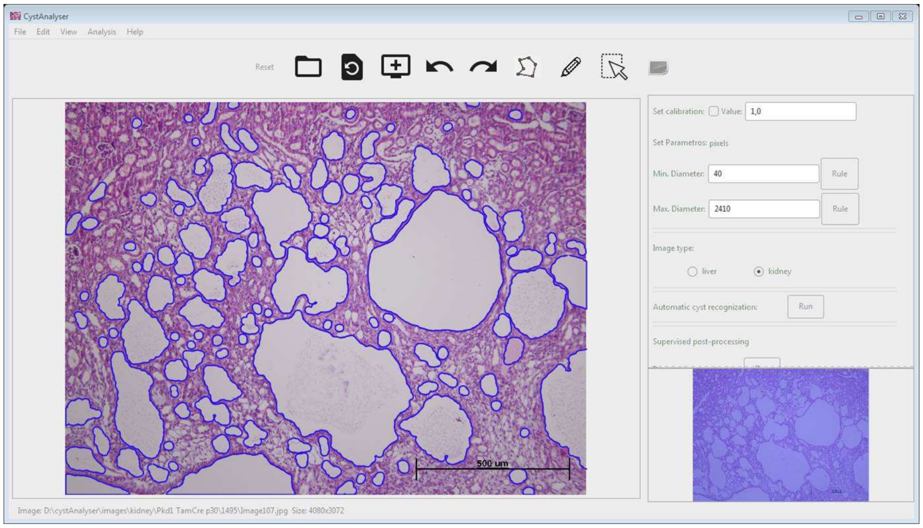

Figure 22: Output of automatically processing a histological image of kidney.

do the following steps: 1) click on the **Rule** button (the button remains activated); 2) draw a line on the image window pressing the left mouse button and when you move the mouse, with the left mouse button pressed, the line is drawing; and 3) when you release the left mouse button, the **Rule** button will become desactivated and the length of the line is put in the entry widget (this value does not appear if the minimum diameter is

superior than maximum diameter or the maximum diameter is lower than the minimum one).

3. **Type of image** (fiveth and sixth lines): the sixth line contains two radio buttons with the labels *Liver* and *Kidney* to select the type of image you are processing. This fact is due to the automatic cyst recognition algorithm is specific of the type of image used. Click on a radio button to select one type of image. The type of image selected does not change until the user modifies it.

4. **Automatic Cyst Recognition** (seventh line): this line only has a **Run** button to automatically process the image (with the preferences set) and show on the image window the cysts recognized by the computer overlapped on the image. Figure 22 shows the result of automatically processing a typical histological image of cystic kidney.

5. **Supervised post-processing**: in this block, there are some tools to help the user to supervise the recognition results of the automatic algorithms. These tools are detailled in the section 8.1.

6. **Visualization of results**: this block includes the following funcionality: 1) press the button **Show Table** to show diameter and area of the cysts overlapped in the image; 2) show the cystic index (after the label *Cystic Index*); 3) show the number of cyst in the image (after the label *Cyst number*); and 4) set the interval to compute the cysts profile. A detailled description of these funcionalities are shown in section 8.2.

7. **Visualization position**: at the bottom of the lateral panel, there is a miniature image of the original image loaded in CystAnalyser. Over this miniature, there is overlapped a blue shadown square showing the part of the original image, which is shown in the image window. The position and size of this blue square depends on the zoom used in that moment, as can be see in figure 20.

8.1 Graphical tools to supervise the cyst recognition.

Once the image was automatically processed using the button **Run** (after the label *Automatic cyst recognition* of the lateral panel), the block of funcionality labeled as *Supervised post-processing* in the lateral panel allows to review the automatic cyst recognition in a friendly and graphical way. The funcionalities are:

1. *Removed inner cyst*: remove the cysts, which fall into a cyst manually drawn. This operation is done when the user clicks the button **Run** after the label *Remove inner cyst*. Figure 23 show an example of this operation: the upper panel show the status before the user clicks that button and the lower panel shows the results of this action.

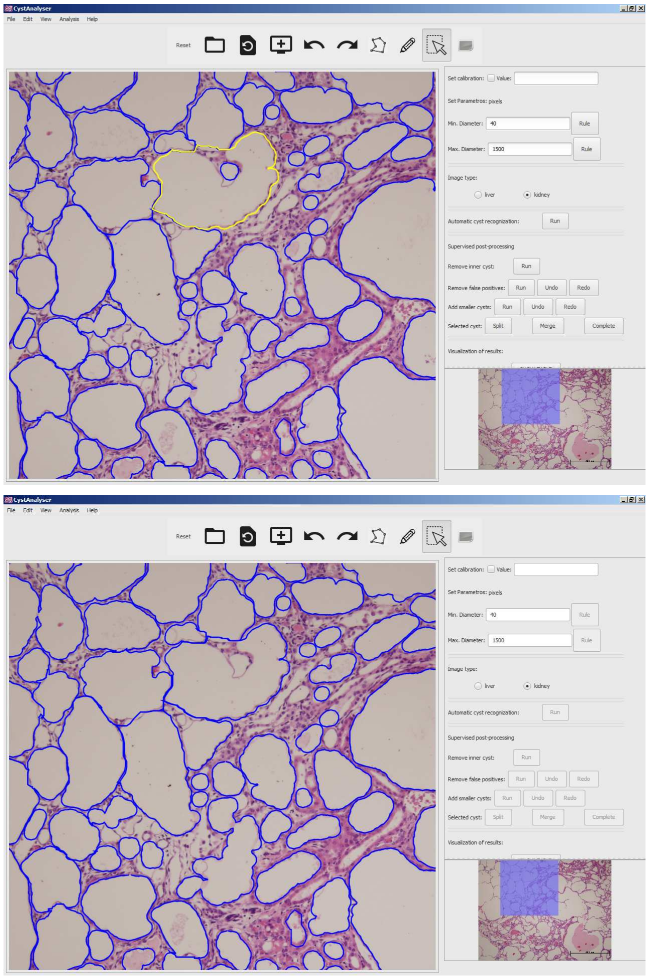

Figure 23: *Remove inner cyst* tool of the lateral panel to remove the cysts, which fall into manually drawn cysts.

2. *Add smaller cyst*: to automatically recognize the smallest cysts in the image. When you click the button **Run** after the label *Add smaller cyst*, CystAnalyser provides the smallest cysts. Figure 24 shows an example of this tool: the upper panel shows the state before using this tool and the lower panel shows the results, in which some of the smallest cysts were added. This operation can be undo and redo using the **Undo** and **Redo** buttons after the label *Add smaller cyst*.

3. *Select cyst*: this label encloses operations to be applyed on one or some selected cysts. The operations, you can apply, are: 1) split one selected

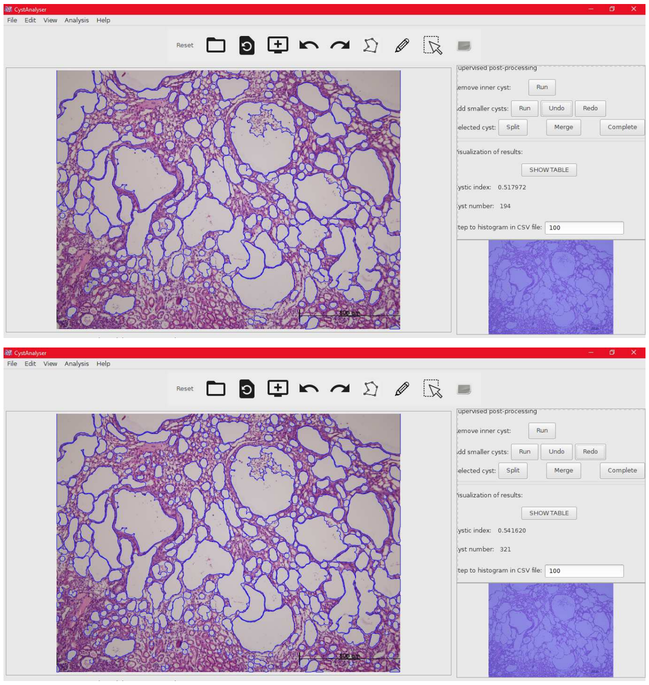

Figure 24: *Add smaller cyst* tool of the lateral panel to supervise the automatic recognized cysts.

cysts into two cysts (button **Split**); 2) merge some selected cysts into one cyst (button **Merge**); and 3) complete one selected cyst with an arc (button **Complete**). To select one cyst, activate the button **Select** (nineth button) in the tool bar and click with the left button of the mouse into the cyst, you want to select. To select more than one cyst, repeat the process keeping the Control key pressed. To do the split operation, you must select the cyst, press the button **Split** (this button will be activated), draw an arc on the image splitting the selected cyst, and, when you finished the drawing of the arc, the **Split** button will be desactivated and the selected cyst will be divided into two cysts. Figure 25 shows two images, representing the state before and after the split operation.

To do the merge operation, you must select the set of cysts you want to merge and click the button **Merge** in the lateral panel. After that, the merged cysts will not be visible and it will appear the new cyst. Figure 26 shows two images representing the state before and after the merge operation.

To do the complete operation, you must select the cyst, which is incomplete in the image, and click the button **Complete** in the lateral panel.

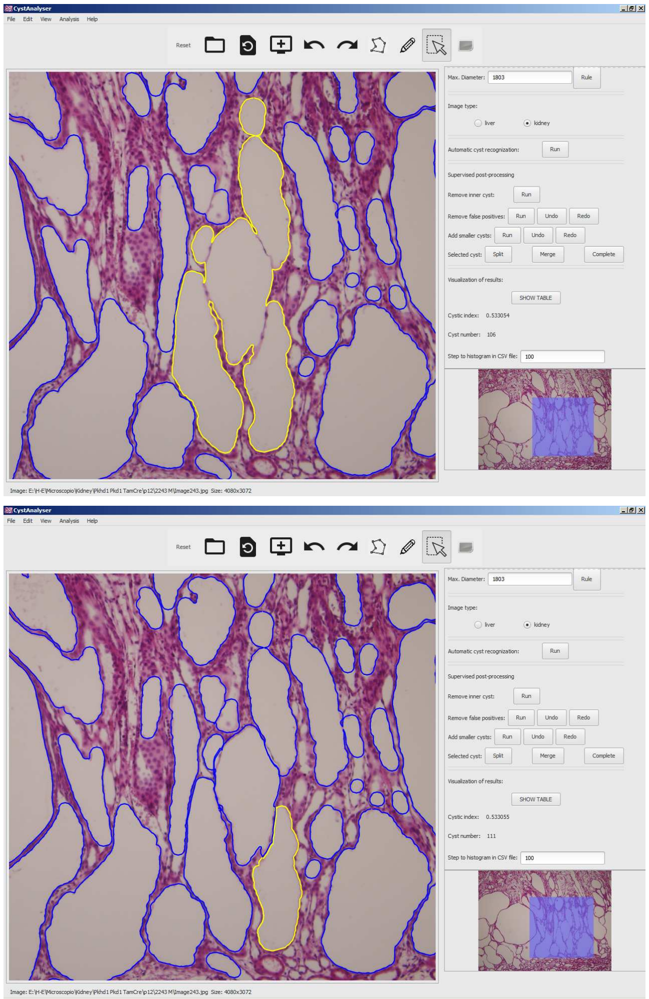

Figure 25: Split operation (button **Split** in the lateral panel) to divide one selected cyst into two cysts.

This button will be activated while the user is drawing an arc on the image to complete the truth outline of the selected cyst. When the user release the left button of the mouse, the selected cyst will be completed using the arc and the **Complete** button will be desactivated. The three images of the Figure 27 show this process.

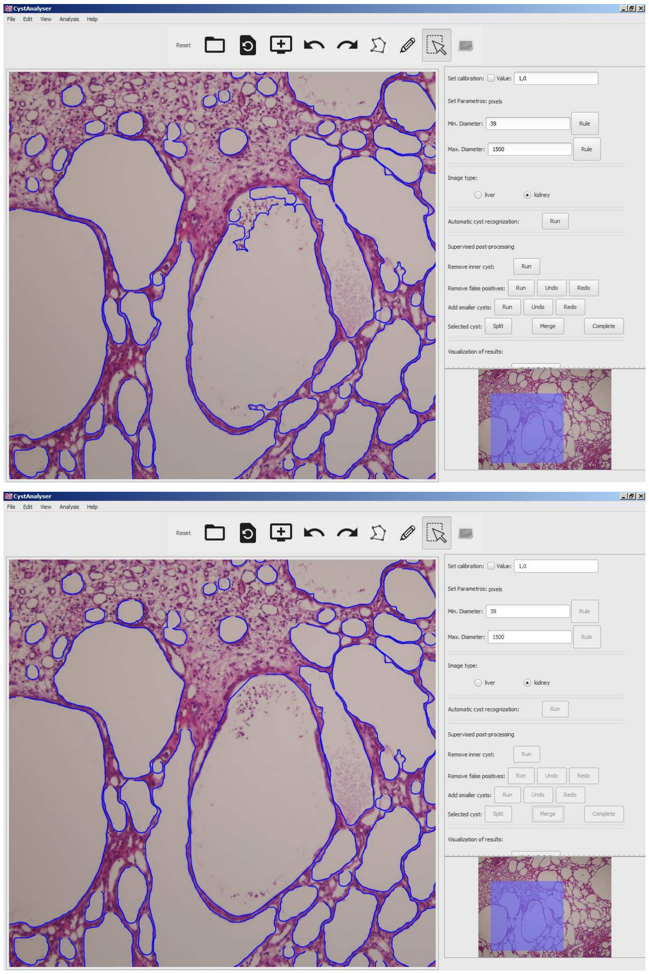

Figure 26: Merge operation (button **Merge** in the lateral panel) to merge various selected cysts into one cyst.

8.2 Visualization of results.

Pressing the button **Show Table** of the lateral panel open a table at the bottom of window (see Figure 28) to show the diameter and the area of the cysts overlapped in the image window. The window image and this table are sincronized. So, if you select a cyst on the image (the objects in the image window can be selected using the nineth icon of tool bar), the row of the table containing its diameter and area will be activated, as it can be seen in the lower panel of Figure 28. In the same way, if you select a row in the table, its corresponding cyst in the image window will be appear as selected, as it can be seen in the

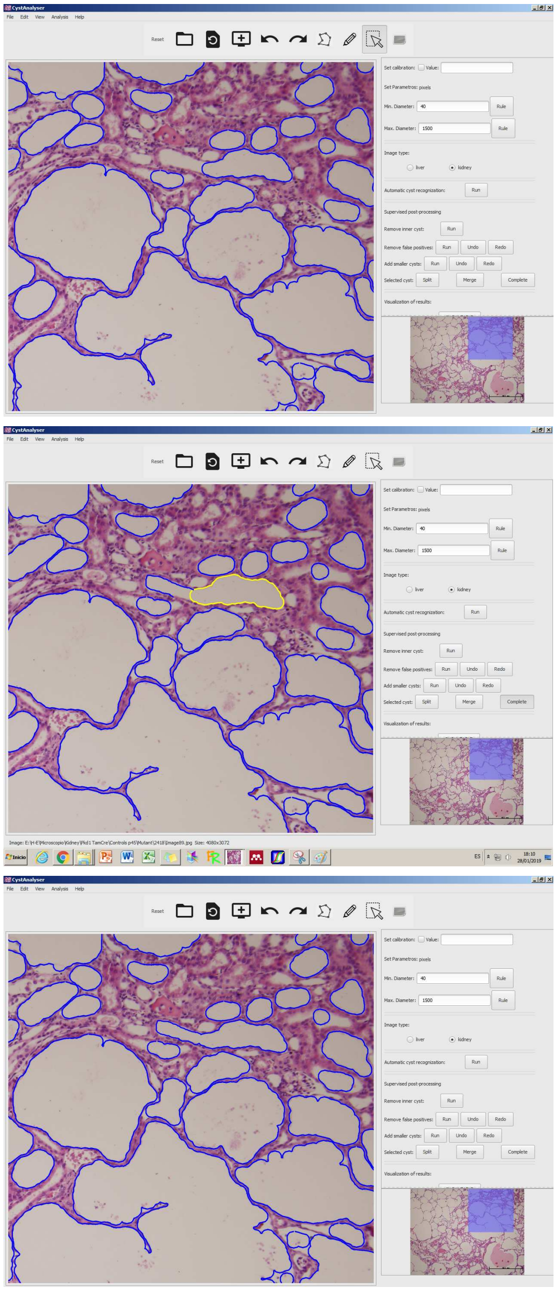

Figure 27: Complete operation (button **Complete** in the lateral panel) to complete the truth outline of the selected cyst.

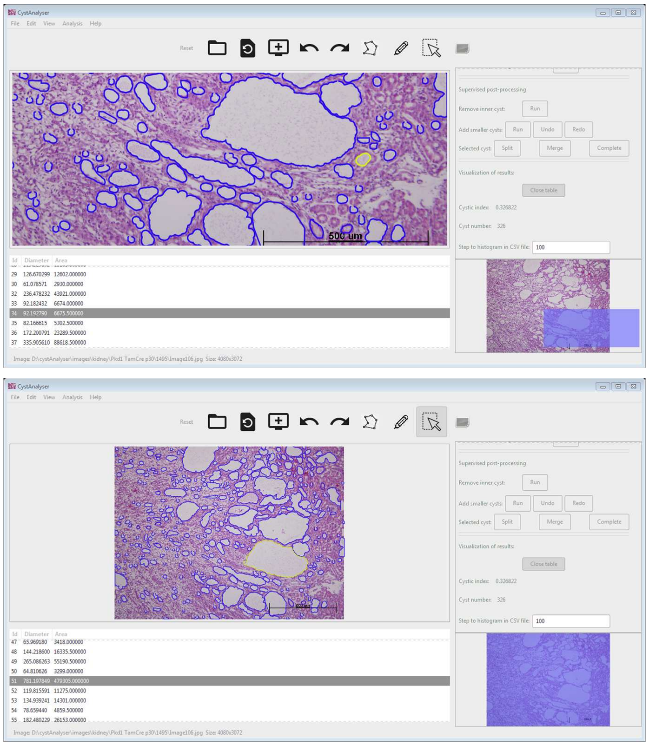

Figure 28: Visualization of the area and diameter of cysts overlapped to the image (button **Show Table** in the lateral panel) .

upper panel of Figure 28. The information shown in this table can be exported to a CSV file, as it has been described in the section 6.

9 Menu Analysis

Figure 29 shows the content of **Analysis** menu, which is in the menu bar. The only item available is **XML File**, which opens the pop up window of figure 30. Manay times the biomedical researchers want to acumulate the quantitative results of various images together. For this purpose, you must do the following steps: 1) process individually each image; 2) save the recognition results (outline of the cysts of each image) in a XML file; and 3) run the submenu **XML File** of menu **Analysis** to calculate the jointly quantitative results.

The first line of window of figure 30, labelled as *XML Folder*, allows to choose the directory of the XML files (by default, it is the XML directory set in the preferences, but it can be changed clicking the three points button). The second line, labelled as *Select XML File*, have the button **Click to select files**.

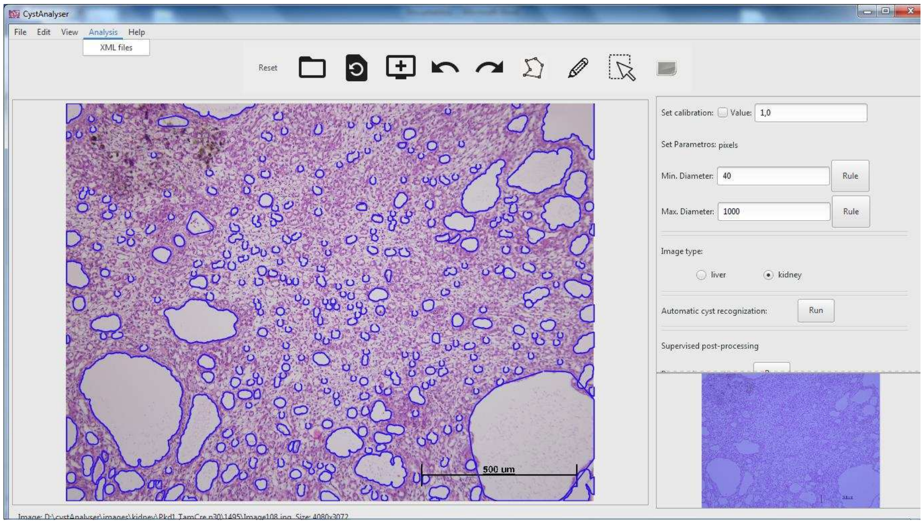

Figure 29: Funcionality available in the **Analysis** menu of CystAnalyser.

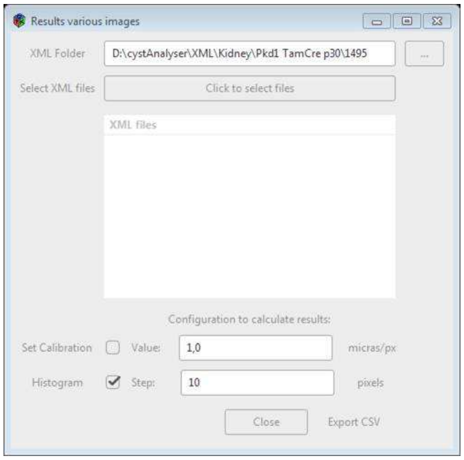

Figure 30: Window open when the submenu **XML File** of menu **Analysis** is chosen.

Clicking this button, the file chooser dialog of figure 31 is open to choose the XML files used to compute the statistics results. As all file chooser dialogs, a file is selected if you click on the file name with the left button mouse. To choose consecutive files, you click the first file and, keeping the key **Alt** pressed, you click the last one. To choose various files, you click files names keeping the key **Ctrl** pressed. Once the XML files were chosen, click the **Select** button in the bottom of the window. The files selected will be appeared in the Figure 30, as it can be seen in the Figure 32.

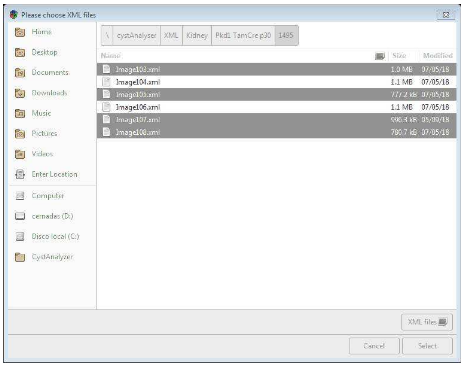

Figure 31: File chooser dialog to choose the XML files included in the jointly analysis.

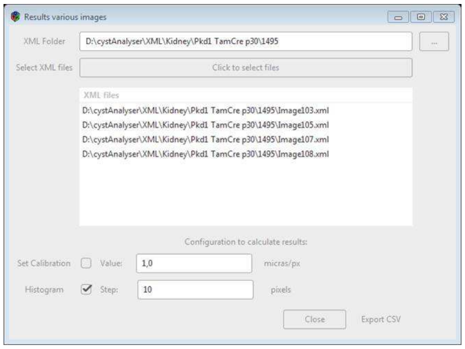

Figure 32: Window open when the submenu **XML File** of menu **Analysis** is chosen, after selecting the XML files used in the analysis.

To complete the parameters used in the joined analysis of Figure 32 (lines under the label *Configuration to calculate results*) , you can select the calibration and histogram step. As in the preferences configuration (section 5), you can choose to activate or not activate the calibration marking the check box after the label *Set Calibration*. If calibration is activated, you can set its value in the entry widget after the label *Value*. The histogram funcionality allows to create cysts profiles, i. e. count the number of cysts in each range of diameters. So, for histogram, you can set to calculate or not calculate the histogram marking the check box after the label *Histogram*. If the histogram calculation is set, the step used can be set in the entry widget after the label *Step*. The value of this step will be considered in pixels, if the calibration is not activated, and in micrometers if the calibration is activated.

After filling all the information in the window of Figure 33, you can choose the following operations: 1) cancel this operation pressing the **Close** button in the bottom; or 2) press the **Export CSV** to store the joined statistical results

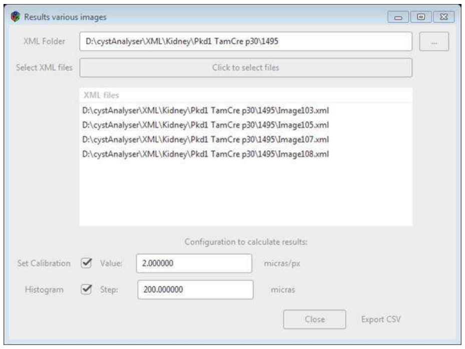

Figure 33: An example of figure 32, in which the user changes the default parameters for the calibration and histogram preferences.

in a CSV file. This last operation open a file chooser dialog, as in Figure 17, to provide the name of the CSV file. This operation could require some seconds if there are many XML files to analyse. After saving one CSV file, the window of Figure 33 goes on open until the user click the **Close** button, in order to do other analysis. On example of the CSV file stored can be observed in the Figure 34.

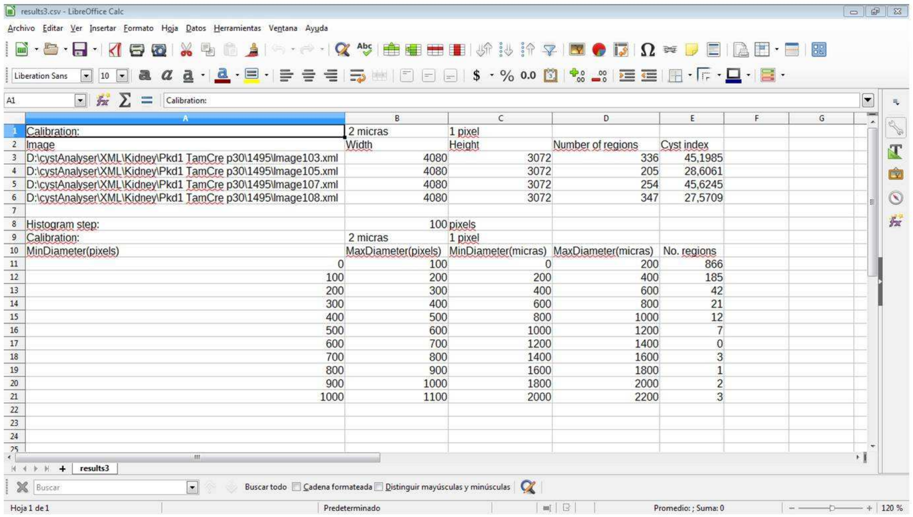

Figure 34: An example of CSV file of the analysis of various XML files imported in LibreOffice Calc.

10 Menu Help

The **Help** menu is within the menu bar (see Figure 35) with the submenus **User Manual** and **About Us**. The submenu **About Us** pop-ups a window with a decription of CystAnalyser and informations about thanks and licence (see Figure 36).

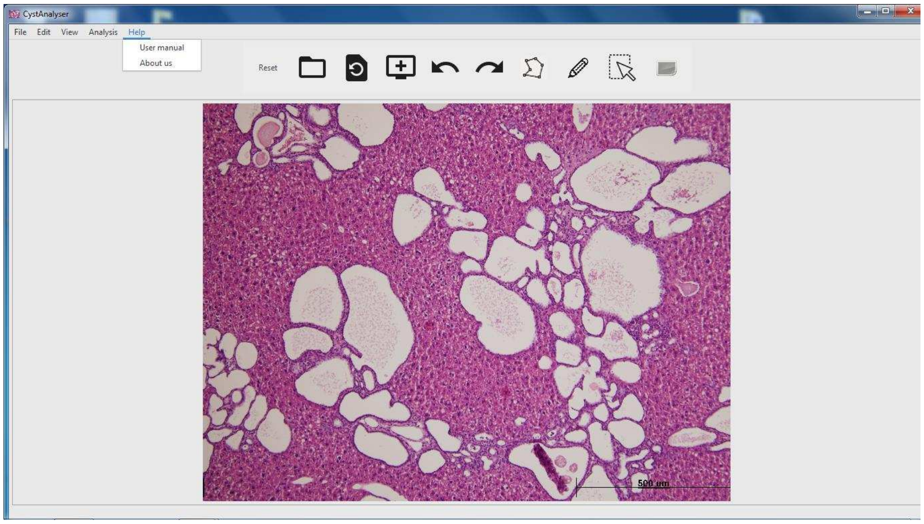

Figure 35: Funcionality available in the Help menu of CystAnalyser.

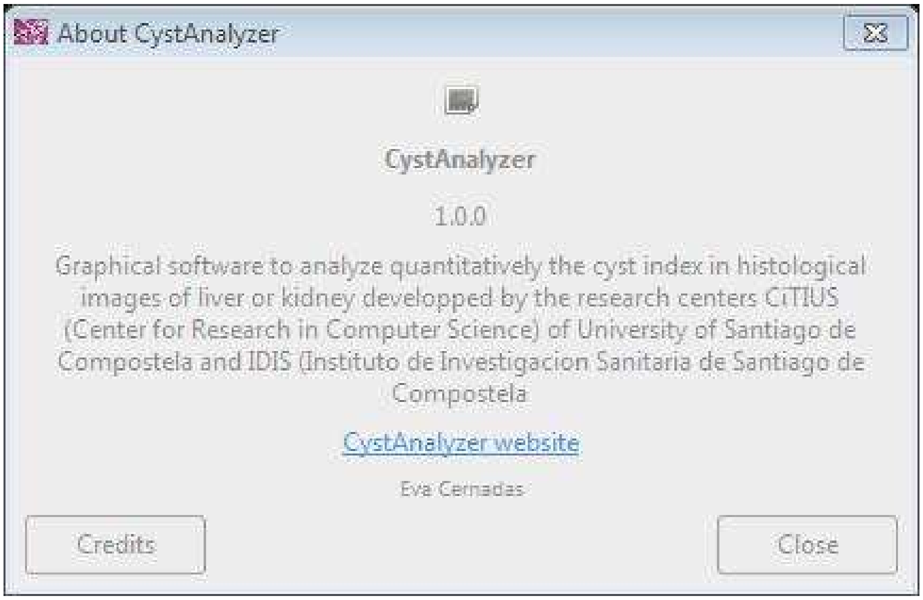

Figure 36: Dialog to inform about help.
